# Predicted Influences of Artificial Intelligence on the Domains of Nursing: Scoping Review

**DOI:** 10.2196/23939

**Published:** 2020-12-17

**Authors:** Christine Buchanan, M Lyndsay Howitt, Rita Wilson, Richard G Booth, Tracie Risling, Megan Bamford

**Affiliations:** 1 Registered Nurses' Association of Ontario Toronto, ON Canada; 2 Arthur Labatt Family School of Nursing Western University London, ON Canada; 3 College of Nursing University of Saskatchewan Saskatoon, SK Canada

**Keywords:** nursing, artificial intelligence, machine learning, robotics, patient-centered care, review

## Abstract

**Background:**

Artificial intelligence (AI) is set to transform the health system, yet little research to date has explored its influence on nurses—the largest group of health professionals. Furthermore, there has been little discussion on how AI will influence the experience of person-centered compassionate care for patients, families, and caregivers.

**Objective:**

This review aims to summarize the extant literature on the emerging trends in health technologies powered by AI and their implications on the following domains of nursing: administration, clinical practice, policy, and research. This review summarizes the findings from 3 research questions, examining how these emerging trends might influence the roles and functions of nurses and compassionate nursing care over the next 10 years and beyond.

**Methods:**

Using an established scoping review methodology, MEDLINE, CINAHL, EMBASE, PsycINFO, Cochrane Database of Systematic Reviews, Cochrane Central, Education Resources Information Center, Scopus, Web of Science, and ProQuest databases were searched. In addition to the electronic database searches, a targeted website search was performed to access relevant gray literature. Abstracts and full-text studies were independently screened by 2 reviewers using prespecified inclusion and exclusion criteria. Included articles focused on nursing and digital health technologies that incorporate AI. Data were charted using structured forms and narratively summarized.

**Results:**

A total of 131 articles were retrieved from the scoping review for the 3 research questions that were the focus of this manuscript (118 from database sources and 13 from targeted websites). Emerging AI technologies discussed in the review included predictive analytics, smart homes, virtual health care assistants, and robots. The results indicated that AI has already begun to influence nursing roles, workflows, and the nurse-patient relationship. In general, robots are not viewed as replacements for nurses. There is a consensus that health technologies powered by AI may have the potential to enhance nursing practice. Consequently, nurses must proactively define how person-centered compassionate care will be preserved in the age of AI.

**Conclusions:**

Nurses have a shared responsibility to influence decisions related to the integration of AI into the health system and to ensure that this change is introduced in a way that is ethical and aligns with core nursing values such as compassionate care. Furthermore, nurses must advocate for patient and nursing involvement in all aspects of the design, implementation, and evaluation of these technologies.

**International Registered Report Identifier (IRRID):**

RR2-10.2196/17490

## Introduction

### Artificial Intelligence

Artificial intelligence (AI) is a branch of computer science that focuses on building machines that can perform tasks that typically require human intelligence, such as decision making, speech recognition, visual perception, and language translation [1]. AI health technologies (AIHTs) are becoming increasingly prevalent in clinical settings worldwide, and global spending on these technologies is predicted to exceed US $36 billion by 2025 [2]. Given their potential to enhance workflows and guide clinical decision making, AIHTs are predicted to directly and indirectly transform the nursing profession in various ways.

Nurses are the largest group of health professionals, and they currently practice in diverse settings and roles across the 5 domains of nursing activity identified by the Registered Nurses’ Association of Ontario (RNAO; ie, administration, education, clinical practice, policy, and research) [3]. Within the nursing profession, the delivery of person- and family-centered compassionate care is a core and valued component of nursing theory and practice [4-6] and is reflected in numerous nursing practice frameworks [7-9]. Compassionate care helps nurses to shift their focus from simply completing tasks to engaging fully with patients by recognizing and responding to their individual needs, promoting well-being, and forming therapeutic relationships essential to effective care [10,11]. It is anticipated that emerging trends in AIHTs will change the nature of the nurse-patient relationship [2]. Therefore, strong nursing leadership is required to drive this change and ensure the continued delivery of high-quality, person-centered compassionate nursing care [12]. Recent studies and expository papers have begun to explore the influence of AIHTs on nursing roles, workflows, processes, and patient care. However, no published scoping reviews have mapped the breadth and depth of evidence concerning the current or predicted influences of AIHTs on the nursing profession and compassionate nursing care. Furthermore, there is limited research on nurses’ roles in influencing the implementation of AIHTs and the co-design of these technologies to protect patients’ safety and privacy and preserve person- and family-centered compassionate care. Without an understanding of the existing evidence on this topic, nurses will not fully appreciate the implications of AI for nursing practice, policy, administration, and research. It is critical for nurses to gain a broader understanding of these emerging technologies to shape the future of the profession and influence decisions about aspects of nursing care that can be safely performed by AIHTs.

### Background

The increased adoption of AIHTs in health care, driven partially by growing consumer demands for digital health technologies in clinical practice [13], may present a new means of addressing health challenges in the 21st century by enhancing workflows and supporting clinical decision making [14]. Over the last few decades, the volume of technology has increased significantly in many health systems around the world, and consequently, the impact of technology on the nursing role has become an increasing focus within nursing research [15]. There is an undeniable relationship that exists between human beings, technology, and the environment [16], and these relationships need to be further examined through the lens of nursing practice to effectively leverage AIHTs to augment the patient experience and health outcomes [17].

Within the nursing profession, many different types of AIHTs are already being used or trialed, including predictive analytics that use machine learning (ML), virtual health care assistant apps, and robotic devices. ML is a subset of AI that uses algorithms to derive knowledge from data and interpret the data without being explicitly programmed [2]. As more data are presented to the ML application, the computer learns from the data and corrects the output [2]. Predictive analytics that use ML technology can identify patterns in data and predict future patient outcomes such as a patient’s risk of developing pressure ulcers [18]. Predictive analytics have been integrated into smart health care technologies to predict health status changes among patients in hospitals and community-based settings, enabling nurses to proactively intervene and initiate appropriate interventions [19]. Nurses in a Canadian home health organization use virtual health care assistant apps (chatbots) to support persons who have been diagnosed as having mental health conditions [20]. Furthermore, in Japan, where approximately 30% of the population is older than 65 years, nurses use AI-powered robots in long-term care (LTC) homes and hospital settings to assist patients with activities of daily living and to provide social interaction [21].

As AIHTs become increasingly prevalent within clinical practice settings, nurses in all roles and across all domains will need to consider the influence of these technologies on the nurse-patient relationship and on the nursing profession more broadly. Furthermore, to ensure that the nursing-AI relationship promotes person-centered compassionate care, it will be important to understand how nurses may contribute to the co-design of AIHTs. This scoping review explores how AIHTs influence the complex relationships between nurses and their patients as well as nursing practice across the domains of clinical practice, policy, administration, and research.

### Objectives

A scoping review was conducted to summarize the findings of 4 distinct research questions that explore the relationships between nurses, patients, and AIHTs [22]. Given the number of articles included in the scoping review, the decision was made to divide the results into 2 stand-alone papers to improve clarity. This review summarizes the findings of 3 of those research questions, specifically exploring the influences of emerging trends in AIHTs on the nurse-patient relationship and the nursing domains (Textbox 1). The fourth research question discusses the influences of emerging trends in AIHTs on nursing education, and the results of that research question were conducive to a stand-alone paper on that topic, which is forthcoming (Buchanan C et al, unpublished data, 2021).

**Research questions addressed in this review.**

The following questions were addressed:

1. What influences do artificial intelligence–driven digital health technologies have, or are predicted to have, on the patient or caregiver experience of compassionate care delivered by nurses?
2. What influences do emerging trends in artificial intelligence–driven digital health technologies have, or are predicted to have, on all domains of nursing practice (ie, administration, clinical care, education, policy, and research)?
3. What involvement do nurses have, or are predicted to have, in the co-design of artificial intelligence–driven digital health technologies?

A scoping review methodology was deemed appropriate for this topic because of the emerging nature of AI in nursing and the exploratory nature of scoping reviews, intended to address broad research questions [23,24]. To the best of the authors’ knowledge, no scoping reviews have mapped existing evidence on the relationship between AI, nursing, and the nurse-patient relationship.

## Methods

### Scoping Review

This scoping review followed the framework developed by Arksey and O’Malley [24] and further advanced by Levac et al [23], which is composed of 6 stages: (1) identifying the research question; (2) identifying relevant studies; (3) study selection; (4) charting the data; (5) collating, summarizing, and reporting the results; and (6) consultation [24]. The scoping review was registered in the Open Science Framework database [25], and a protocol outlining the full methods can be found published elsewhere [22]. This reporting of the scoping review is guided by the Preferred Reporting Items for Systematic Reviews and Meta-Analyses extension for Scoping Reviews (PRISMA-ScR) checklist [26].

### Steering Committee Formation

A 14-member steering committee was established to identify the research questions, inform the search strategy, and provide consultation throughout the scoping review [22]. The committee was cochaired by 2 doctorally prepared nurses with independent research programs focused on health informatics and the integration of AI and digital health technologies in nursing (RB and TR). All committee members had considerable knowledge or expertise with the use or implementation of AI or digital health technologies in nursing or the health system. The members included a patient advocate with lived experience and nurses from a variety of settings (eg, hospital care, home and community care, and LTC), domains (eg, administration, clinical practice, research, education, and policy), and roles (eg, nurse executives, nurse informaticians, nurse managers, professional practice leaders, and frontline nurses).

### Identifying the Research Questions

The research questions and search strategy were developed through consultation with the steering committee. The latter was refined with the assistance of an information specialist at a large academic teaching hospital library (Multimedia Appendix 1). Using a Boolean combination of keywords and medical subject headings, MEDLINE, CINAHL, EMBASE, PsycINFO, Cochrane Database of Systematic Reviews, Cochrane Central, Education Resources Information Center, Scopus, Web of Science, and ProQuest were searched for peer-reviewed literature published between January 1, 2014, and October 17, 2019, which focused on AI and nursing [22]. A targeted website search was also performed to locate the relevant gray literature. Google search strings were developed by the information specialist, and the following websites were searched: World Health Organization, National Health Service, Office of the National Coordinator for Health Information Technology, Institute for Research on Healthy Public Policy, Canada Health Infoway, Canadian Association of Schools of Nursing, and Healthcare Information and Management Systems Society.

### Identifying Relevant Studies

A screening guide was developed by the 2 reviewers (CB and LH), and 2 levels of screening were performed using predefined inclusion and exclusion criteria [22]. During title and abstract screening, articles were independently assessed and included if they were deemed relevant to the concepts of *AI* and *nursing* [22]. Next, full-text papers were independently reviewed and assessed for their relevance to 1 of the 4 research questions [22]. Conflicts were resolved through discussion, and when a disagreement arose, a third independent reviewer (RW) assessed whether the publication met the inclusion criteria [22].

### Study Selection

To be included for question 1, articles had to have a clear focus on AIHTs, health professionals, and caring. Although the authors initially planned to only include articles relevant to AIHTs, nursing, and compassionate care, there was a paucity of literature regarding the impact of AI on compassionate care and nursing specifically. Consequently, the inclusion criteria were refined after consultation with the cochairs. Articles that discussed AIHTs, health professionals, and caring were used to address question 1, and the findings were generalized to nursing and compassionate care. The reviewers agreed on a clear definition of *compassionate care* before screening, defined as an empathetic response that involves engaging fully with patients by recognizing and responding to their individual needs, promoting well-being, and forming therapeutic relationships essential to care [10,11]. Articles that discussed *care* that pertained to this definition were included whether or not the article explicitly used the term *compassionate care*. Similarly, articles that referred to health professionals in general were also included if the information was relevant to nursing practice, even if there was no explicit reference to nurses [22]. To be included for question 2, articles had to address how AIHTs are currently influencing or are predicted to influence the domains of nursing as outlined by the RNAO [3], specifically, clinical practice, policy, administration, and research [3]. Finally, for question 3, articles had to discuss how nurses have been involved in the co-design of AIHTs or what nurses can contribute to the design of these technologies.

### Charting the Data

Standardized data charting forms for each research question were created by the 2 reviewers and tested with a representative sample of articles [22]. Once it was determined that consistency in data charting was achieved between the reviewers, data from each included article was charted by one reviewer and verified by the second reviewer [22]. Information on the author, year, study design, country, aim/purpose, population, type of AIHT discussed, key findings related to each research question, and relevance to compassionate care was charted. Findings were recorded by study type in separate data charting forms for each research question (ie, qualitative vs quantitative study designs and expository papers).

### Collating, Summarizing, and Reporting the Results

An inductive approach was used to categorically summarize the findings from the included papers to answer each research question [22]. Each paper was reviewed several times, and CB, LH, and RW discussed the findings and came to a consensus on identifying categories for each research question based on commonalities observed. The findings from quantitative and qualitative articles as well as expository or review papers were analyzed as a collective group of research. The categories were then summarized in the form of a data package and sent to members of the steering committee for review.

### Consultation

Overall, 2 virtual steering committee meetings were held to provide feedback on the categories and implications of the findings. As stated by Levac et al [23], consultation should be an essential component of the scoping review methodology, and preliminary findings (ie, the scoping review categories identified) can be used as a foundation to inform the consultation. Levac et al [23] have suggested that consultation allows for knowledge transfer and exchange with stakeholders in the field, and stakeholders can offer additional information, perspectives, meaning, and applicability to the scoping review. Thus, the virtual steering committee meetings allowed for discussion of the findings and categories with the committee members, and feedback was received regarding how the identified categories may be applicable or relevant to clinical practice, policy, administration, and research.

## Results

### Overview of Studies

Overall, 131 articles were retrieved from the scoping review for the 3 research questions (118 from database sources and 13 from targeted websites; Multimedia Appendix 2). Specifically, there were 51 articles pertaining to research question 1, 98 articles pertaining to research question 2, and 16 articles pertaining to research question 3; however, it is important to note that some articles addressed multiple research questions. The results included all types of studies (quantitative, qualitative, mixed method, systematic reviews, and scoping reviews) as well as gray literature including reports, editorials, and opinion pieces (Multimedia Appendix 3 provides more details [2,13,14,18,21,27-152]). A total of 16 included articles were also cited in the systematic and scoping reviews that were included.

### Emerging Trends in AIHTs

Emerging AIHTs discussed in the literature included robots (eg, socially assistive robots [SARs], humanoid robots, and mobility robots), predictive analytics, clinical decision support systems (CDSSs), smart homes, and virtual health care assistant chatbots. It was found that nurses are already using robots in their clinical practice, across multiple patient populations, for various tasks, such as to assist with exercise sessions for older adults or rehabilitation patients [27,28]; to serve as a distraction tool for pain management [29-31]; and to facilitate conversation and rapport, conduct interviews, and deliver patient education [27,33,34,36-39, 41, 42].

The literature also discussed life-like virtual health care assistant chatbots that have been used in clinical practice settings to support nursing practice and provide persons seeking internet-based mental health care with information about resources and additional support [43]. One article discussed advancements in sensory and processing developments that provide virtual health care assistant chatbots with greater capabilities, enabling them to detect, interpret, and express emotions as well as detect other behavioral signals of humans they interact with (eg, facial gestures and body posture/movement) [43].

### Research Question 1: Nursing, Compassionate Care, and AI

Although there is consensus in the literature that emerging trends in AIHTs will impact nursing care [2,44], there was a paucity of literature on the potential impacts of AIHTs on compassionate nursing care specifically. Consequently, articles that discussed AIHTs and caring were used to address this research question, and the findings were generalized to compassionate care. The vast majority of included articles examined the influence of robots on the delivery of care (Multimedia Appendix 3).

Multiple articles predicted that by using robots to assist with some care activities, nurses may have more time to spend in getting to know their patients’ preferences, responding appropriately to their needs, and building stronger therapeutic relationships [35,45-48]. In addition, several articles discussed how health professionals, including nurses, have used SARs to gain a deeper understanding of their patients [34,49]. For instance, within LTC settings, SARs have been used to stimulate memories of residents with dementia, allowing health professionals to explore the residents’ past experiences, personality, and identity [34,50,51]. Health professionals have also used SARs to provide emotional support and reduce loneliness among older adults in aged care facilities [36,52] and provide comfort to patients at the end of life [34]. Using SARs during activities with residents may increase the level of engagement between health professionals and residents as they participate in these interactions together [51]. In one case study and one qualitative descriptive study, health professionals and students noted that the use of SARs has led to deeper relationships and increased rapport between health professionals and residents [28,34].

Other articles provided anecdotal evidence suggesting that AIHTs could potentially have detrimental effects [34,52-57]. When health professionals used SARs with residents in LTC, some patients have become agitated and others have become distressed when nurses have taken the robot away from residents [52,53]. If nurses use robotic devices to monitor adherence to treatment, one article suggested that this has the potential to cause embarrassment or anger among patients, weakening the therapeutic relationship and creating tension [54]. In addition, using robots with patients was viewed as infantilizing older adults [34,55,56]. Several other articles also noted that health professionals such as nurses could be considered guilty of intentionally deceiving patients if the patients are not able to recognize that the robot is not real [53,56,57]. Authors writing from this perspective suggest that AIHTs may negatively impact the patient and caregiver experience of caring and are apprehensive about the potential effect of this technology on nurses’ ability to engage in caring interactions with patients [34,52-57].

### Research Question 2: Implications of AI on Clinical Practice, Administration, and Policy

AI has potential implications on various established domains of nursing. The results of this question and their implications on the domains of clinical practice, administration, and policy are outlined in the following sections.

#### Clinical Practice

There is a consensus among authors that AIHTs will not directly replace nurses in the near future [2,13,14,48,58]. However, it is envisioned that there will be new nurse-patient interactions involving AIHTs in clinical practice that may augment nursing practice and the delivery of safe, high-quality care [14]. These emerging nursing-AI interactions will necessitate the reconceptualization of nursing practice, resulting in new nursing roles, new virtual care delivery models, and new workflows [2,48,59]. In addition, there will likely be increased demand for virtual health care assistant chatbots to support remote patient monitoring and virtual models of care [42,59,60].

It is also reported that AIHTs have the potential to streamline workflow processes and improve the accuracy and efficiency of care provided in diverse clinical settings [14]. Specifically, it is presumed that AIHTs have the potential to decrease nurses’ physical and cognitive workload [61]. A few articles reported that robotic devices and other AIHTs can be used to collect demographic and health-related information, including patient histories, allowing nurses more time for patient care [31,62,63].

In the LTC sector, there is evidence that robotic devices have been used to assist nurses in meeting residents’ hygiene and care needs (eg, toileting, lifting/transferring, and meal delivery), demonstrating the potential for these tools to reduce nursing workload in this clinical setting [21,64-67]. AIHTs such as CDSSs have also been used by nurses in this sector to tailor the residents’ plan of care based on their preferences for activities and personal care, thereby enhancing person-centered care and maximizing the staff’s time [68].

In the hospital sector, AIHTs such as predictive algorithms and CDSSs have been shown to improve decision making and nursing activities (eg, documentation), allowing more time for patient care [69-76]. For example, one study noted that a predictive algorithm used for the identification of nursing diagnoses reduced the time spent on decision making from 35.5 min to 19.8 min [61]. Multiple articles also noted that predictive algorithms can assist nurses with faster detection of patient changes and more efficient timely care [77-79].

#### Administration

The literature reviewed identified many applications of AIHTs in administration. For example, AIHTs may be used to schedule nursing tasks and assign patients to rooms [66]. AIHTs may also help to reduce the documentation burden [80] and assist with the scheduling of patient appointments [73]. One article suggested that modifications to the administration and scheduling of staff in LTC settings are needed to accommodate the emerging AIHTs [81]. It is also predicted that some administrative tasks that are currently performed solely by regulated staff will become more complex and require some automation [14,82]. Furthermore, new administration roles for nurse leaders are predicted to emerge [13]. One article predicted that nurses may act as case and information navigators [13] using AIHTs to assist in organizing and prioritizing patient care transitions through the health system.

#### Policy

Several authors suggested that with the integration of AI, new policies will be necessary to address concerns related to patient safety and ethical practice [14,43]. One such concern relates to unintended consequences associated with the use of AIHTs that might potentially impact patients’ health and well-being [43]. For example, patients may be harmed because of technology failures or malfunctioning of robots [37]. Furthermore, without adequate protection of data, patient privacy could be jeopardized [35]. In addition, there were concerns in the literature about the use of predictive analytics to guide clinical decision making. Unlike other risk prediction tools that show how risk scores are calculated, when using predictive analytics, it is not always possible to identify the factors used in the clinical assessment and determination of risk [58]. Therefore, who would be deemed responsible if an error were to occur was not clear [43].

The importance of identifying and addressing real or potential health inequities related to AIHTs was also noted in the literature [62]. For instance, although these technologies have the potential to enhance access to care and health service delivery, they may also accentuate health inequities by increasing the digital divide [62]. Due diligence is required to ensure that vulnerable populations and people in rural and remote areas have access to continuous coordinated care [62]. In reality, people in rural and remote areas may have a greater need for virtual care and remote patient monitoring than people living in urban areas [83].

### Research Question 3: Nursing Research and Co-Design of AIHTs

Although 2 articles noted that nurse researchers are in a natural position to contribute to the research on and development of AIHTs to ensure a smooth transition from development to clinical practice [70,84], this scoping review found minimal information regarding the current involvement of nurses in the research and co-design of these technologies.

In addition, 2 articles mentioned examples of projects where nurses partnered with engineers and programmers to develop robots [2,85]; however, there was no explanation of the specific roles and responsibilities nurses assumed while contributing to these projects. One study noted that although the involvement of nurses in the conceptual, research, and developmental stages of any new product or system is integral, nurses are often involved in testing or evaluating a product when major functional changes to the technology may not be possible [85]. Although nurses are frequently employed by engineering companies in the sales or marketing of products, they are less involved in research and development activities [85].

The literature also described several barriers that could influence the involvement of nurses in the co-design process and other barriers that may impede the implementation of these technologies. Communication barriers may arise because of nurses and information technology (IT) experts being unfamiliar with each other’s field of practice [86]. In addition, nurses may not be familiar with the technical terms used by IT experts, and IT experts may not understand nursing concepts. In one study, both groups learned to overcome communication barriers by avoiding the use of technical jargon and by meeting frequently to reduce communication errors [86]. At an organizational level, the cost of hiring doctorally prepared nurses to lead innovative projects and a lack of organizational support for the inclusion of nurses in a data science strategy may also prevent involvement in co-design [70].

Multiple articles discussed the skills, knowledge, and expertise that nurses can contribute to the co-design of AIHTs [2,63,85-87]. Nurses uniquely understand the complexities of the health care environment [63], including what works, what does not work, and what can be done to improve patient experiences and health outcomes [85]. Given the key role nurses play in supporting the physical and psychological health of persons and families, nurses are in a unique position to advocate for the needs and preferences of patients [87] and identify the ways patients are best served by technology [88].

## Discussion

### Overview

The principal aim of this review is to summarize the findings of 3 research questions that explored emerging trends in AIHTs and their potential influence on the roles and functions of nurses and the delivery of compassionate care over the next 10 years and beyond. The implications of the findings for clinical practice, policy, administration, and research are discussed in more detail in the following sections.

### Nursing Practice

As mentioned previously, there is a dearth of literature on the topic of compassionate care, nursing, and AI. Most of the included articles focused on robots and caring within health care in general, and there were mixed findings about the potential influence of AIHTs on the delivery of compassionate nursing care.

The integration of AIHTs into health organizations will have significant implications for nurses across all domains of practice. Implications for clinical practice were reported in the literature, with some articles discussing the potential for robots to reduce nursing workload by assisting with activities of daily living [21,64-67]. One potential unintended consequence of this emerging trend is the concern that nurses may actually spend less time with their patients or be given a larger patient workload as a result. Therefore, the implementation of these AIHTs in any practice setting must be closely monitored to ensure that nurses and other health professionals use them to augment and enhance care and not replace it.

Strong and proactive nursing leadership in all roles, sectors, and domains will be required to effectively implement these technologies in ways that preserve person-centered compassionate care. The new and evolving nursing care delivery models that are envisioned will require strong leadership from nurse executives, who will play a significant leadership role in identifying the requisite competencies for direct care providers who will be using AIHTs in practice.

New opportunities may arise for nurses to work as care coordinators, using AIHTs such as robots to assist with patient care activities or as case managers who remotely monitor a caseload of patients using smart home technology [13]. Nurses have been encouraged to imagine the possibilities that can be actualized through the convergence of nursing, technology, and caring [64,153]. Several articles in this review suggested new possibilities for the delivery of compassionate nursing care. For instance, using robotic devices to assist nurses may result in patients having their needs met in a timelier manner and the enrichment of the nurse-patient relationship [34,46-48]. Furthermore, in LTC settings, health professionals have reportedly used SARs to enhance resident care by reducing loneliness and improving mood among persons diagnosed as having dementia [36,55] and to provide comfort to patients at the end of life [34].

However, concerns have been raised that the use of robots in clinical practice is deceptive for some patients [57], creates tension [54], infantilizes older adults [34,55,56], and may promote culturally insensitive care [89], which could serve to weaken the therapeutic relationships among nurses and those they care for. Given these mixed findings, it is crucial that nurses understand the needs and preferences of residents, families, and caregivers before introducing SARs, and they must continuously monitor how the resident is responding to the robot [34,55]. Clarifying expectations about robots has been suggested as a means of preventing deception [53].

Finally, the delivery of person-centered compassionate care necessitates cultural sensitivity, and nurses must recognize the inability of robots to recognize cultural cues and respond appropriately [89]. It may be useful to hold forums for nurse leaders that will stimulate discussion about the best implementation approaches. This approach could help ensure that AIHTs are integrated into nursing practice in a way that aligns with compassionate care and other nursing values.

### Nursing Policy

The results of this review identified the need to develop new policies to support the integration of AIHTs into nursing practice in ways that will promote patient safety and high-quality care. The integration of AI in nursing will require strong leadership to develop new policies and procedures to support new models of care, new nursing roles, new workflows, and potential changes to the nursing scope of practice. For example, it will be necessary for regulatory bodies to develop or revise standards of practice that articulate nurses’ accountabilities for clinical judgment and decision making when using predictive analytics. To address these concerns, professional codes of ethics and standards of practice will require clear stipulations that the use of digital health technologies such as AI are intended to augment rather than replace nurses’ clinical judgment [2,90]. In the United States, the American Nurses Association’s *Code of Ethics for Nurses with Interpretive Statements* has begun to address this concern by stating, “Systems and technologies that assist in clinical practice are adjunct to, not replacements for, the nurse’s knowledge and skill” [154]. In addition, policies are needed to promote patient safety and ethical practice because patients are at risk for harm related to technology failures [37], and privacy breaches could jeopardize patient information [35]. Finally, to ensure AIHTs do not accentuate health inequities, policy makers will need to ensure that there is adequate telecommunication infrastructure in remote areas so that AIHTs can be used in these settings [62].

### Nursing Administration

The results of the review found numerous implications for nursing administration. Some articles discussed the use of AIHTs to assist nurses with automated analysis of patient data [69-76]. Other administration applications of AIHTs included scheduling nursing tasks [66], reducing documentation burden [80], and assisting nurses with triaging patients through AIHT computer systems [14], which could potentially aid in streamlining workflow processes and improving the efficiency and accuracy of patient care provided. It is also predicted that the integration of AI in nursing will give rise to new nursing care delivery models and new nursing administration roles [48].

### Nursing Research

The paucity of literature has shown that the study of AI and compassionate care is still in its infancy, and more research on this topic is required. In addition, given that most research conducted on the topic of AIHTs and caring has focused on the use of robots, future research should explore how other types of AIHTs may influence the delivery of compassionate care. As AIHTs become increasingly integrated into nursing practice, it is also essential that increased support and funding is provided to identify best practices for optimal implementation across the care continuum. In collaboration with other health disciplines, nurses are in an ideal position to lead research on AI and compassionate care, given that compassionate care is a core tenet of the nursing profession.

Several authors have described the clinical and research expertise nurses can bring to the co-design of AIHTs [70,84]; however, this scoping review found minimal information regarding the current involvement of nurses in co-design. Our findings align with those of other authors who have noted the paucity of information regarding how nurses are involved in the co-design of AIHTs [91,92]. Given the valuable contributions nurses can bring to the co-design of these technologies, nurse executives should advocate for the establishment of clinical nursing informatics officers in health organizations to guide the procurement, design, and implementation of AIHTs. Increased nursing input in the co-design of AIHTs will help ensure that these technologies serve to enhance the delivery of compassionate care and not hinder it. Furthermore, nursing leadership will be needed at both the executive and staff nurse levels to promote nursing and patient engagement in co-design activities focused on AIHTs.

### Limitations

Several limitations must be considered when interpreting the findings. Because of accessibility issues and organizational licensing restrictions, computer science and engineering databases were not searched for this scoping review, which may have led to research gaps. It is recommended that future reviews on the topic of AI and nursing use these databases. Non-English papers were excluded from this review, and the reference lists of included studies were not searched. Thus, it is likely that this review missed some relevant articles. In addition, as mentioned previously, for research question 1, the reviewers generalized the findings from articles that discussed *nursing and caring* to *nursing and compassionate care* because of the paucity of literature specifically pertaining to compassionate care. This approach uses an element of reviewer judgment while screening articles and may have influenced the final articles included in the review. Finally, the reviewers did not use Cohen kappa to calculate interrater agreement during title and abstract screening and instead used a percentage agreement (97% agreement). Although this was done for feasibility purposes, it is recognized that percentage agreement is not as reliable as Cohen kappa.

### Conclusions

AI has already begun to shape nursing roles, workflows, and the nurse-patient relationship. To our knowledge, this is the first scoping review to have mapped the breadth and depth of evidence concerning the current and predicted influences of AI technologies on compassionate care and the domains of nursing. With the anticipated growth of AI in nursing, the findings of this review will help nurse leaders at all levels and across all sectors to proactively shape the nursing-AI interface, ensuring alignment with core nursing values that promote ethical, safe, high-quality, and person-centered compassionate care for patients, families, and caregivers.

## Appendix

Multimedia Appendix 1 MEDLINE search strategy.

Multimedia Appendix 2 PRISMA (Preferred Reporting Items for Systematic Reviews and Meta-Analyses) diagram.

Multimedia Appendix 3 Table of article characteristics.

## Abbreviations

AI: artificial intelligence
AIHT: artificial intelligence health technology
AMS: Associated Medical Services
CDSS: clinical decision support system
CEO: chief executive officer
IT: information technology
LTC: long-term care
ML: machine learning
RNAO: Registered Nurses’ Association of Ontario
SAR: socially assistive robot

## References

[1] Lexico (2019). Artificial intelligence. Lexico US Dictionary.

[2] Robert N (2019). How artificial intelligence is changing nursing. Nurs Manage.

[3] Registered Nurses' Association of Ontario (2007). Professionalism in nursing.

[4] Gaut D, Leninger M (1991). Caring: The Compassionate Healer.

[5] Curtis K (2015). Compassion is an essential component of good nursing care and can be conveyed through the smallest actions. Evid Based Nurs.

[6] Chambers C, Ryder E (2009). Compassion and Caring In Nursing.

[7] Canadian Nurses Association (2015). Framework for the practice of registered nurses in Canada.

[8] Australian Nursing and Midwifery Federation (2018). Nursing practice.

[9] American Nurses Association (2015). Nursing scope and standards of practice (3rd edition).

[10] Sharp S, McAllister M, Broadbent M (2016). The vital blend of clinical competence and compassion: How patients experience person-centred care. Contemp Nurse.

[11] Perez-Bret E, Altisent R, Rocafort J (2016). Definition of compassion in healthcare: a systematic literature review. Int J Palliat Nurs.

[12] Associated Medical Services (AMS) Healthcare (2018). Compassion in a technological world: Advancing AMS' strategic aims.

[13] Booth R (2016). Informatics and Nursing in a Post-Nursing Informatics World: Future Directions for Nurses in an Automated, Artificially Intelligent, Social-Networked Healthcare Environment. Nurs Leadersh.

[14] Secretary of State for Health and Social Care (2019). The Topol review: Preparing the health care workforce to deliver the digital future.

[15] Booth GB, Andrusyszyn M, Iwasiw C, Donelle L, Compeau D (2016). Actor-Network Theory as a sociotechnical lens to explore the relationship of nurses and technology in practice: methodological considerations for nursing research. Nurs Inq.

[16] Ihde D (1990). Technology And The Lifeworld: From Garden To Earth (Indiana Series In The Philosophy Of Technology).

[17] Watson D, Womack J, Papadakos S (2020). Rise of the robots: is artificial intelligence a friend or foe to nursing practice?. Crit Care Nurs Q.

[18] Cramer EM, Seneviratne MG, Sharifi H, Ozturk A, Hernandez-Boussard T (2019). Predicting the incidence of pressure ulcers in the intensive care unit using machine learning. EGEMS (Wash DC).

[19] Chawla N (2020). AI, IOT and wearable technology for smart healthcare? A review. Int J Green Energy.

[20] Gionet K (2018). Meet Tess: the mental health chatbot that thinks like a therapist.

[21] Maalouf N, Sidaoui A, Elhajj IH, Asmar D (2018). Robotics in Nursing: a scoping review. J Nurs Scholarsh.

[22] Buchanan C, Howitt ML, Wilson R, Booth RG, Risling T, Bamford M (2020). Nursing in the age of artificial intelligence: protocol for a scoping review. JMIR Res Protoc.

[23] Levac D, Colquhoun H, O'Brien KK (2010). Scoping studies: advancing the methodology. Implement Sci.

[24] Arksey H, O'Malley L (2005). Scoping studies: towards a methodological framework. Int J Soc Res Methodol.

[25] Buchanan C, Howitt ML, Bamford M (2020). Nursing and Compassionate Care in the Age of Artificial Intelligence: A Scoping Review (Registration). Open Science Framework Registries.

[26] Tricco AC, Lillie E, Zarin W, O'Brien KK, Colquhoun H, Levac D, Moher D, Peters MD, Horsley T, Weeks L, Hempel S, Akl EA, Chang C, McGowan J, Stewart L, Hartling L, Aldcroft A, Wilson MG, Garritty C, Lewin S, Godfrey CM, Macdonald MT, Langlois EV, Soares-Weiser K, Moriarty J, Clifford T, Tunçalp Özge, Straus SE (2018). PRISMA extension for scoping reviews (PRISMA-ScR): checklist and explanation. Ann Intern Med.

[27] Ujike S, Yasuhara Y, Osaka K, Sato M, Catangui E, Edo S, Takigawa E, Mifune Y, Tanioka T, Mifune K (2019). Encounter of Pepper-CPGE for the elderly and patients with schizophrenia: an innovative strategy to improve patient's recreation, rehabilitation, and communication. J Med Invest.

[28] Tuisku O, Pekkarinen S, Hennala L, Melkas H (2019). 'Robots do not replace a nurse with a beating heart': the publicity around a robotic innovation in elderly care. ITP.

[29] Demange M, Pino M, Kerhervé H, Rigaud A, Cantegreil-Kallen I (2019). Management of acute pain in dementia: a feasibility study of a robot-assisted intervention. J Pain Res.

[30] Jibb L, Birnie K, Nathan P, Beran T, Hum V, Victor J, Stinson J (2018). Using the MEDiPORT humanoid robot to reduce procedural pain and distress in children with cancer: a pilot randomized controlled trial. Pediatr Blood Cancer.

[31] Beran T, Ramirez-Serrano A, Vanderkooi O, Kuhn S (2015). Humanoid robotics in health care: an exploration of children's and parents' emotional reactions. J Health Psychol.

[32] Nieto Franco F (2018). Design and implementation of a robotic software for the assessment of pain in children. Stud Health Technol Inform.

[33] Tanioka R, Sugimoto H, Yasuhara Y, Ito H, Osaka K, Zhao Y, Kai Y, Locsin R, Tanioka T (2019). Characteristics of transactive relationship phenomena among older adults, care workers as intermediaries, and the pepper robot with care prevention gymnastics exercises. J Med Invest.

[34] Birks M, Bodak M, Barlas J, Harwood J, Pether M (2016). Robotic seals as therapeutic tools in an aged care facility: a qualitative study. J Aging Res.

[35] Liang H, Wu K, Weng C, Hsieh H (2019). Nurses' views on the potential use of robots in the pediatric unit. J Pediatr Nurs.

[36] Robinson H, Broadbent E, MacDonald B (2016). Group sessions with paro in a nursing home: structure, observations and interviews. Australas J Ageing.

[37] Huisman C, Kort H (2019). Two-year use of care robot Zora in Dutch nursing homes: an evaluation study. Healthcare (Basel).

[38] Bemelmans R, Gelderblom G, Jonker P, de Witte L (2016). How to use robot interventions in intramural psychogeriatric care; a feasibility study. Appl Nurs Res.

[39] Boumans R, van Meulen F, Hindriks K, Neerincx M, Olde Rikkert MG (2019). Robot for health data acquisition among older adults: a pilot randomised controlled cross-over trial. BMJ Qual Saf.

[40] Jones C, Moyle W, Murfield J, Draper B, Shum D, Beattie E, Thalib L (2018). Does cognitive impairment and agitation in dementia influence intervention effectiveness? Findings from a cluster-randomized-controlled trial with the therapeutic robot, Paro. J Am Med Dir Assoc.

[41] Sicurella T, Fitzsimmons V (2016). Robotic pet therapy in long-term care. Nursing.

[42] Miller E, Polson D (2019). Apps, avatars, and robots: the future of mental healthcare. Issues Ment Health Nurs.

[43] Luxton DD (2014). Recommendations for the ethical use and design of artificial intelligent care providers. Artif Intell Med.

[44] Tanioka T, Yasuhara Y, Dino MJ, Kai Y, Locsin RC, Schoenhofer SO (2019). Disruptive engagements with technologies, robotics, and caring: advancing the transactive relationship theory of nursing. Nurs Adm Q.

[45] Beck J (2019). Are we ready for AI? Why innovation in tech needs to be matched by investment in people. Eurohealth.

[46] Kriegel J, Grabner V, Tuttle-Weidinger L, Ehrenmüller I (2019). Socially assistive robots (SAR) in in-patient care for the elderly. Stud Health Technol Inform.

[47] Vandemeulebroucke T, Dierckx de Casterlé B, Welbergen L, Massart M, Gastmans C (2020). The ethics of socially assistive robots in aged care. A focus group study with older adults in flanders, belgium. J Gerontol B Psychol Sci Soc Sci.

[48] Pepito JA, Locsin R (2019). Can nurses remain relevant in a technologically advanced future?. Int J Nurs Sci.

[49] Tanioka T (2019). Nursing and rehabilitative care of the elderly using humanoid robots. J Med Invest.

[50] Gustafsson C, Svanberg C, Müllersdorf M (2015). Using a robotic cat in dementia care: a pilot study. J Gerontol Nurs.

[51] Pfadenhauer M, Dukat C (2015). Robot caregiver or robot-supported caregiving?. Int J of Soc Robotics.

[52] Moyle W, Jones C, Murfield J, Thalib L, Beattie E, Shum D, Draper B (2019). Using a therapeutic companion robot for dementia symptoms in long-term care: reflections from a cluster-RCT. Aging Ment Health.

[53] Vandemeulebroucke T, Dierckx de Casterlé B, Gastmans C (2018). The use of care robots in aged care: a systematic review of argument-based ethics literature. Arch Gerontol Geriatr.

[54] Zafrani O, Nimrod G (2019). Towards a holistic approach to studying human-robot interaction in later life. Gerontologist.

[55] Moyle W, Bramble M, Jones C, Murfield J (2018). Care staff perceptions of a social robot called Paro and a look-alike Plush Toy: a descriptive qualitative approach. Aging Ment Health.

[56] Vandemeulebroucke T, de Casterlé BD, Gastmans C (2018). How do older adults experience and perceive socially assistive robots in aged care: a systematic review of qualitative evidence. Aging Ment Health.

[57] Metzler T, Lewis L, Pope L (2016). Could robots become authentic companions in nursing care?. Nurs Philos.

[58] Lynn L (2019). Artificial intelligence systems for complex decision-making in acute care medicine: a review. Patient Saf Surg.

[59] Ackerman M, Virani T, Billings B (2017). Digital mental health - innovations in consumer driven care. Nurs Leadersh.

[60] Joerin A, Rauws M, Ackerman M (2019). Psychological artificial intelligence service, Tess: delivering on-demand support to patients and their caregivers: technical report. Cureus.

[61] Liao P, Hsu P, Chu W, Chu W (2015). Applying artificial intelligence technology to support decision-making in nursing: a case study in Taiwan. Health Informatics J.

[62] Kuziemsky C, Maeder AJ, John O, Gogia SB, Basu A, Meher S, Ito M (2019). Role of artificial intelligence within the telehealth domain. Yearb Med Inform.

[63] Clipper B, Batcheller J, Thomaz AL, Rozga A (2018). Artificial Intelligence and Robotics: a nurse leader's primer. Nurse Leader.

[64] Archibald M, Barnard A (2018). Futurism in nursing: technology, robotics and the fundamentals of care. J Clin Nurs.

[65] Papadopoulos I, Koulouglioti C, Ali S (2018). Views of nurses and other health and social care workers on the use of assistive humanoid and animal-like robots in health and social care: a scoping review. Contemp Nurse.

[66] Glauser W (2017). Artificial intelligence, automation and the future of nursing. Can Nurse.

[67] Ding M, Matsubara T, Funaki Y, Ikeura R, Mukai T, Ogasawara T (2017). Generation of comfortable lifting motion for a human transfer assistant robot. Int J Intell Robot Appl.

[68] Gannod G, Abbott K, Van Haitsma K, Martindale N, Heppner A (2019). A machine learning recommender system to tailor preference assessments to enhance person-centered care among nursing home residents. Gerontologist.

[69] McGrow K (2019). Artificial intelligence: essentials for nursing. Nursing.

[70] Linnen D, Javed P, DʼAlfonso JN (2019). Ripe for disruption? Adopting nurse-led data science and artificial intelligence to predict and reduce hospital-acquired outcomes in the learning health system. Nurs Adm Q.

[71] Olling K, Nyeng DW, Wee L (2018). Predicting acute odynophagia during lung cancer radiotherapy using observations derived from patient-centred nursing care. Tech Innov Patient Support Radiat Oncol.

[72] Mandal I (2016). Machine learning algorithms for the creation of clinical healthcare enterprise systems. Enterp Inf Syst.

[73] (2018). The Topol review interim report: Preparing the health care workforce to deliver the digital future. NHS Health Education England.

[74] Healthcare Information and Management Systems Society (HIMSS) (2019). Artificial intelligence, critical thinking and the nursing process.

[75] Healthcare Information and Management Systems Society (HIMSS) (2018). AI and nursing impact on the quadruple aim.

[76] Joshi I, Morley J (2019). Artificial Intelligence: How to get it right. Putting policy into practice for safe data-driven innovation in health and care. NHSX.

[77] Li H, Lin SW, Hwang YT (2019). Using nursing information and data mining to explore the factors that predict pressure injuries for patients at the end of life. Comput Inform Nurs.

[78] Sensmeier J (2017). Harnessing the power of artificial intelligence. Nurs Manage.

[79] Davoudi A, Malhotra K, Shickel B, Siegel S, Williams S, Ruppert M, Bihorac E, Ozrazgat-Baslanti T, Tighe P, Bihorac A, Rashidi P (2019). Intelligent ICU for autonomous patient monitoring using pervasive sensing and deep learning. Sci Rep.

[80] Byrne MD (2017). Machine learning in health care. J Perianesth Nurs.

[81] Salzmann-Erikson M, Eriksson H (2017). Letter to the editor: prosperity of nursing care robots: an imperative for the development of new infrastructure and competence for health professions in geriatric care. J Nurs Manag.

[82] Foley T, Wollard J (2019). The digital future of mental healthcare and its workforce: A report on a mental health stakeholder engagement to inform the Topol Review. NHS Health Education England.

[83] Fritz R, Corbett C, Vandermause R, Cook D (2015). The influence of culture on older adults' adoption of smart home monitoring. Gerontechnology.

[84] Fritz R, Dermody G (2019). A nurse-driven method for developing artificial intelligence in 'smart' homes for aging-in-place. Nurs Outlook.

[85] Glasgow M, Colbert A, Viator J, Cavanagh S (2018). The nurse-engineer: a new role to improve nurse technology interface and patient care device innovations. J Nurs Scholarsh.

[86] Shorey S, Ang E, Yap J, Ng ED, Lau ST, Chui CK (2019). A virtual counseling application using artificial intelligence for communication skills training in nursing education: development study. J Med Internet Res.

[87] Backonja U, Hall A, Painter I, Kneale L, Lazar A, Cakmak M, Thompson J, Demiris G (2018). Comfort and attitudes towards robots among young, middle-aged, and older adults: a cross-sectional study. J Nurs Scholarsh.

[88] Risling T (2018). Why AI needs nursing. Policy Options.

[89] Papadopoulos I, Koulouglioti C (2018). The influence of culture on attitudes towards humanoid and animal-like robots: an integrative review. J Nurs Scholarsh.

[90] Carroll WM (2019). The synthesis of nursing knowledge and predictive analytics. Nurs Manage.

[91] Risling T, Low C (2019). Advocating for safe, quality and just care: what nursing leaders need to know about artificial intelligence in healthcare delivery. Nurs Leadersh (Tor Ont).

[92] Dermody G, Fritz R (2019). A conceptual framework for clinicians working with artificial intelligence and health-assistive Smart Homes. Nurs Inq.

[93] Klein B, Schlömer I (2018). A robotic shower system : acceptance and ethical issues. Z Gerontol Geriatr.

[94] Fiorini L, De Mul M, Fabbricotti I, Limosani R, Vitanza A, D'Onofrio G, Tsui M, Sancarlo D, Giuliani F, Greco A, Guiot D, Senges E, Cavallo F (2019). Assistive robots to improve the independent living of older persons: results from a needs study. Disabil Rehabil Assist Technol.

[95] Law M, Sutherland C, Ahn HS, MacDonald BA, Peri K, Johanson DL, Vajsakovic D, Kerse N, Broadbent E (2019). Developing assistive robots for people with mild cognitive impairment and mild dementia: a qualitative study with older adults and experts in aged care. BMJ Open.

[96] Poulsen A, Burmeister OK (2019). Overcoming carer shortages with care robots: dynamic value trade-offs in run-time. Australas J Inf Sys.

[97] Lee H, Piao M, Lee J, Byun A, Kim J (2020). The purpose of bedside robots: exploring the needs of inpatients and healthcare professionals. Comput Inform Nurs.

[98] Coco K, Kangasniemi M, Rantanen T (2018). Care personnel's attitudes and fears toward care robots in elderly care: a comparison of data from the care personnel in Finland and Japan. J Nurs Scholarsh.

[99] Rantanen T, Lehto P, Vuorinen P, Coco K (2018). The adoption of care robots in home care-A survey on the attitudes of Finnish home care personnel. J Clin Nurs.

[100] Bemelmans R, Gelderblom G, Jonker P, de Witte Luc (2015). Effectiveness of robot Paro in intramural psychogeriatric care: a multicenter quasi-experimental study. J Am Med Dir Assoc.

[101] Chen TL, King CA, Thomaz AL, Kemp CC (2013). An investigation of responses to robot-initiated touch in a nursing context. Int J of Soc Robotics.

[102] de Fátima Fernandes FM, Esteves RB, Teixeira CA, da Silva Gherardi-Dona EC (2018). The present and the future of nursing in the brave new world. Rev Esc Enferm USP.

[103] Nwosu A, Collins B, Mason S (2018). Big Data analysis to improve care for people living with serious illness: the potential to use new emerging technology in palliative care. Palliat Med.

[104] Moyle W, Jones C, Pu L, Chen SC (2018). Applying user-centred research design and evidence to develop and guide the use of technologies, including robots, in aged care. Contemp Nurse.

[105] Nairn S (2016). On being a Luddite in the new world of technological nursing care. Nurs Philos.

[106] Whelton B (2016). Being human in a global age of technology. Nurs Philos.

[107] Monteiro A (2016). Cyborgs, biotechnologies, and informatics in health care-new paradigms in nursing sciences. Nurs Philos.

[108] Newland J (2015). Humans versus artificial intelligence. Nurse Pract.

[109] Hernandez JP (2019). Network diffusion and technology acceptance of a nurse chatbot for chronic disease self-management support : a theoretical perspective. J Med Invest.

[110] Gallagher A, Nåden D, Karterud D (2016). Robots in elder care: some ethical questions. Nurs Ethics.

[111] Digital Futures. Alder Hey Children's NHS Foundation Trust.

[112] Clavelle J, Sweeney C, Swartwout E, Lefton C, Guney S (2019). Leveraging technology to sustain extraordinary care: a qualitative analysis of meaningful nurse recognition. J Nurs Adm.

[113] Poncette A, Spies C, Mosch L, Schieler M, Weber-Carstens S, Krampe H, Balzer F (2019). Clinical requirements of future patient monitoring in the intensive care unit: qualitative study. JMIR Med Inform.

[114] Morley J, Joshi I (2019). Developing effective policy to support artificial intelligence in health and care. Eurohealth: digital health systems.

[115] Carroll W (2018). Predicting severe maternal morbidity and mortality - an informatics opportunity. Online J Nurs Inform.

[116] Carroll W (2019). Nursing informaticists safeguarding the use of emerging technologies. Online J Nurs Inform.

[117] Kaminski J (2018). Exploring the nationwide interoperability roadmap from a nursing perspective. Online J Nurs Inform.

[118] Meetoo D, Rylance R (2018). AI: revolution or apocalypse?. Br J Nurs.

[119] Carter-Templeton H, Frazier R, Wu L, H Wyatt T (2018). Robotics in nursing: a bibliometric analysis. J Nurs Scholarsh.

[120] Sherry J (2018). The robot nurses are coming to a workplace near you. Br J Nurs.

[121] Ganapathy K, Abdul S, Nursetyo A (2018). Artificial intelligence in neurosciences: a clinician's perspective. Neurol India.

[122] Skiba D (2017). Augmented intelligence and nursing. Nurs Educ Perspect.

[123] Paulson S, Scruth E (2017). Legal and ethical concerns of big data: predictive analytics. Clin Nurse Spec.

[124] Ishiguro K, Majima Y (2016). Utilization of communication robot in patient education. Stud Health Technol Inform.

[125] Carrière R, MacDonald A, Chan Y (2016). Past, present and future: the outlook from mid-career nurse informaticians. Nurs Leadersh (Tor Ont).

[126] Sharts-Hopko N (2014). The coming revolution in personal care robotics: what does it mean for nurses?. Nurs Adm Q.

[127] Frazier R, Carter-Templeton H, Wyatt T, Wu L (2019). Current trends in robotics in nursing patents? A glimpse into emerging innovations. Comput Inform Nurs.

[128] Peirce AG, Elie S, George A, Gold M, O'Hara Kim, Rose-Facey W (2020). Knowledge development, technology and questions of nursing ethics. Nurs Ethics.

[129] Perry L (2019). Machine learning: Great opportunities, but will it replace nurses?. Int J Nurs Pract.

[130] Woods J, Saxena M, Nagamine T, Howell R, Criscitelli T, Gorenstein S, M Gillette B (2018). The future of data-driven wound care. AORN J.

[131] Nguyen-Truong CKY, Fritz RL (2018). Health-assistive smart homes for aging in place: leading the way for integration of the Asian immigrant minority voice. Asian Pac Isl Nurs J.

[132] Kim J (2018). Use of robots as a creative approach in healthcare ICT. Healthc Inform Res.

[133] Effken JA (2014). Issues, impacts and insights column: What's new in healthcare robotics?. Online Journal of Nursing Informatics.

[134] Jamieson T, Goldfarb A (2019). Clinical considerations when applying machine learning to decision-support tasks versus automation. BMJ Qual Saf.

[135] Lo Y, Lynch S, Urbanowicz R, Olson R, Ritter A, Whitehouse C, O'Connor M, Keim SK, McDonald M, Moore JH, Bowles KH (2019). Using machine learning on home health care assessments to predict fall risk. Stud Health Technol Inform.

[136] Kwon J, Karim M, Topaz M, Currie L (2019). Nurses 'seeing forest for the trees' in the age of machine learning: using nursing knowledge to improve relevance and performance. Comput Inform Nurs.

[137] Guidi G, Pollonini L, Dacso C, Iadanza E (2015). A multi-layer monitoring system for clinical management of Congestive Heart Failure. BMC Med Inform Decis Mak.

[138] Sikka K, Ahmed A, Diaz D, Goodwin M, Craig K, Bartlett M, Huang JS (2015). Automated assessment of children's postoperative pain using computer vision. Pediatrics.

[139] Lin H, Hsu YL, Hsu MS, Cheng CM (2014). Development of a telehealthcare decision support system for patients discharged from the hospital. Telemed J E Health.

[140] Ongenae F, Claeys M, Kerckhove W, Dupont T, Verhoeve P, De Turck F (2014). A self-learning nurse call system. Comput Biol Med.

[141] Park J, Bliss D, Chi C, Delaney C, Westra B (2020). Knowledge discovery with machine learning for hospital-acquired catheter-associated urinary tract infections. Comput Inform Nurs.

[142] Ginestra JC, Giannini HM, Schweickert WD, Meadows L, Lynch MJ, Pavan K, Chivers CJ, Draugelis M, Donnelly PJ, Fuchs BD, Umscheid CA (2019). Clinician perception of a machine learning-based early warning system designed to predict severe sepsis and septic shock. Crit Care Med.

[143] Sullivan SS, Hewner S, Chandola V, Westra BL (2019). Mortality risk in homebound older adults predicted from routinely collected nursing data. Nurs Res.

[144] Lee J, Song YA, Jung JY, Kim HJ, Kim BR, Do H, Lim J (2018). Nurses' needs for care robots in integrated nursing care services. J Adv Nurs.

[145] Turja T, Van Aerschot L, Särkikoski T, Oksanen A (2018). Finnish healthcare professionals' attitudes towards robots: reflections on a population sample. Nurs Open.

[146] Lodhi M, Stifter J, Yao Y, Ansari R, Kee-Nan GM, Wilkie D, Khokhar A (2015). Predictive modeling for end-of-life pain outcome using electronic health records. Adv Data Min.

[147] Sparks RS, Okugami C (2016). Tele-health monitoring of patient wellness. J Intell Syst.

[148] Nauta J, Mahieu C, Michiels C, Ongenae F, De Backere F, De Turck F, Khaluf Y, Simoens P (2019). Pro-active positioning of a social robot intervening upon behavioral disturbances of persons with dementia in a smart nursing home. Cogn Syst Res.

[149] Coahran M, Hillier L, Van Bussel L, Black E, Churchyard R, Gutmanis I, Ioannou Y, Michael K, Ross T, Mihailidis A (2018). Automated fall detection technology in inpatient geriatric psychiatry: nurses' perceptions and lessons learned. Can J Aging.

[150] Menon U, Cohn E, Downs C, Gephart S, Redwine L (2019). Precision health research and implementation reviewed through the conNECT framework. Nurs Outlook.

[151] Frith K (2019). Artificial intelligence: what does it mean for nursing?. Nurs Educ Perspect.

[152] Locsin R (2017). The co-existence of technology and caring in the theory of technological competency as caring in nursing. J Med Invest.

[153] (2018). The Future of Nurses: Superheroes Aided By Technology. The Medical Futurist.

[154] American Nurses Association (2015). Code of ethics for nurses with interpretive statements (2nd ed).

