# Supplementary material for: Predicted Influences of Artificial Intelligence on the Domains of Nursing: Scoping Review
Source: JMIR Nurs. 2020 Dec 17;3(1):e23939. doi: 10.2196/23939 (PMC8373374; doi:10.2196/23939)
Supplement: Multimedia Appendix 2 [file nursing_v3i1e23939_app2.docx]

**Article Review process Flow Diagram**

Records excluded

(n=11,933)

**Identification**

## tion fication

Additional records identified by steering committee
(n = 0)

Records identified by targeted website searches
(n = 2,340)

Records identified through database searching
(n = 19,067)

Records after duplicates removed
(n = 12,658)

(Unable to de-duplicate targeted websites due to technological issues)

**Screening**

## Screening

Records screened for relevance to AI and Nursing

(title and abstract)
(n = 12,658)

**Eligibility**

Full-text records excluded:

- Did not focus on AI technology
- Did not focus on compassionate nursing care, domains of nursing, or nurses involved in the co-design of AI technologies
- Article inaccessible

(n = 530)

Full-text records assessed for relevance to research questions 1, 2, & 3
(n = 661)

Final includes

(total n=131)

(118 database sources, 13 targeted websites)

Question 1: n=51

Question 2: n=98

Question 3: n=16

*Note: some articles were relevant to multiple research questions.*

**Included**

## Included

Flow diagram adapted from D. Moher, A. Liberati, J. Tetzlaff, D. G. Altman, and The PRISMA Group (2009). Preferred Reporting Items for Systematic Reviews and Meta-Analyses: The PRISMA Statement. *BMJ 339*, b2535, doi: 10.1136/bmj.b2535
