# Supplementary material for: Predicted Influences of Artificial Intelligence on the Domains of Nursing: Scoping Review
Source: JMIR Nurs. 2020 Dec 17;3(1):e23939. doi: 10.2196/23939 (PMC8373374; doi:10.2196/23939)
Supplement: Multimedia Appendix 3 [file nursing_v3i1e23939_app3.docx]

| **Research Question** | **Number and Types of Articles** | **Countries** | **Settings** | **Population** | **Types of AI Health Technologies (AIHTs) Discussed** |
| --- | --- | --- | --- | --- | --- |
| What influences do AI-driven digital health technologies have, or are predicted to have, on the patient or caregiver experience of compassionate care delivered by nurses? | -Total of 51 articles for this question  -12 qualitative studies[6,12-14,25, 29,30, 33,71-74]  -6 studies with quantitative or prototyping methods [46,65,76-79]  -2 mixed methods studies [28,75]  -31 expository or review papers (including scoping reviews, editorials and white papers) [2,3,19-24, 26, 27, 31,32,34,35,37,42,43,51,60,67,80-89,131] | Australia [12,20,30,33,42,74,82]  Austria[24]  Belgium [25,31,34]  Brazil [80]  Canada [2,37]  Finland [6,76,77]  Germany [23,29,71]  Israel [32]  Italy [72]  Japan [22,27,131]  Korea[75]  Netherlands [78]  New Zealand [14,73]  Philippines [26]  Portugal [85]  Saudi Arabia [87]  Sweden [28]  Taiwan [13]  United Kingdom [3,43,51,60,67,81,83,88,89]  United States of America [19,21,35,46,65,79,84, 86] | -The majority of articles discussed AIHTs utilized in long-term care (LTC) homes or residential settings for older adults [6,12,14,19,24,28-31,33, 34,46,71-74,76, 78, 82,88]    -Several articles discussed hospital settings [6,13,75,89], community /home care settings [25,37,72,77,87]  or rehabilitation settings [27,76]  -One article discussed a ‘simulated hospital room’ at a robotics lab [79]  -In some articles, settings were not specified or the article discussed multiple settings [2,3,20-23,26,32,35,42, 43, 51,60,65,67,80, 81,83,84, 85,86,131] | -Articles discussed all classes and categories of nurses, most did not specify nursing designations [2,6,13,14,19,22,24,26-28,33, 35,37, 42,43, 65,67,72-78,80,83-86, 131]  -Several articles discussed other types of health professionals (eg, recreational therapists, clinicians, health care workers), but the findings described the influence of technology on the provision of care, and results were applicable to nurses [3,12,20,21,23,29,31,46,51,60,71,81,82,88,89]  -The majority of articles discussed use of AIHTs with older adults [6,12,14,19,24,25,27,28-34,46,71-74,76-78,82,88]  -Two articles discussed pediatric patient populations [13,89]  -One article discussed palliative care patients[81]  -A few articles discussed persons with mental health issues [21,37,60,87] | -The majority of articles described the use of robots (eg, companion robots, socially assistive robots [SARs], humanoid robots, mobility robots)  [6,12-14,19-22,24-35,42, 43,65, 67, 71-79,82,84-86,88,131]  -Several articles described the use of predictive analytics or AI -driven virtual health care assistant chatbots  [20,21,26,37,46,81,87]    -Some articles discussed AIHTs in general [2,3,23,51,60,80,83,89] |
| What influences do emerging trends in AI-driven digital health technologies have, or are predicted to have, on all domains of nursing? | -Total of 98 articles for this question  -12 qualitative studies  [6,11-16, 61, 72, 90-92]  -30 studies with quantitative or prototyping methods (including case studies, cross-sectional designs, retrospective studies) [4, 8-10, 17, 18, 39, 45, 46, 50, 55, 57, 62, 76-78, 114-127]  -4 mixed-methods studies [7, 49, 75, 128]  -52 expository papers (including scoping reviews, editorials and white papers)  [1-3,5, 19-21,26,36-38, 40-44, 47, 48, 51-54, 56, 58-60, 68, 69, 87,89,94-113, 129] | Australia [12,18, 20,42, 108, 126]  Belgium [119,127]  Canada [2,8, 9, 37,38, 40, 44, 69, 104, 113, 128]  Denmark [49]  Finland [6,76,77,124]  France [7]  Germany [91]  India [50, 100]  Italy [72]  Japan [11, 45, 92, 103]  Korea [75, 111, 123]  Lebanon [5]  Netherlands [78, 15-17]  New Zealand [14]  Philippines [26]  Saudi Arabia[87]  Spain [10]  Sweden [59]  Taiwan [13, 39, 55, 118]  United Kingdom [3,43,51,54, 60,89,93, 97, 99]  United States of America [1, 4, 19,21,36, 41, 46-48, 52, 53, 56-58, 61, 62, 68, 90, 94-96, 98, 101, 102, 105-107, 109, 110, 112, 114-117, 120-122, 125, 129] | -Majority of the articles discussed hospital settings [4, 6, 8,9,13,36, 39, 41, 48, 49, 57, 58, 75,90-92, 115-117, 119-121,123,125,128] or LTC settings/residential settings for older adults  [6,7, 12,14-16,18,19,46,59, 72,76,78,127]  -Some articles discussed community/home care  [17, 37,38, 61, 62, 72,76,77,87, 110, 114, 116,122,124,126], rehabilitation settings [11,76], telehealth settings [40, 118]  - In some articles, settings were not specified or the article discussed multiple settings [1-3,5, 10, 20,21,26,42-45,47,50-56,60,68,69,89,93-109, 111-113,129] | -The articles discussed all types of nurses, most did not specify nursing designations  [1,2,4-6,7-10, 13,14,17,19, 26,36,37,39,41-45, 47-50, 52, 53,55-59,61,62,68,69,72,75-78,90-92,94-99,101-113,115-126,128,129]  -Some articles referred to health professionals more generally, but the information was relevant and transferable to nurses [3,11,12,15,16,18, 20,21,38,40,46,51,54, 60,89, 93, 100, 114, 127]  -The articles discussed patients across the continuum of care (ie, pediatrics[8-10, 13,89,117], adults [38,75,87,91,92, 94], and older adults [6,7,11,12,14,15-19, 19,46,59, 61, 62,72,76-78,103, 110, 122, 123, 127] | A variety of AIHTs were described in the articles, including:  -Various types of robotics (eg. service robots, companion robots, SARs, care robots, etc.) [5, 6- 21,26,41-45,59, 72,75-78,92, 98,99,103, 105,106,111,112, 123,124, 127]  -Clinical decision support systems, assessment/diagnosis systems, early warning systems, or predictive analytics that utilize machine learning algorithms [4, 26,36, 39, 41,46-50, 52,53, 55, 58, 68, 90, 91, 94, 102, 109,113-118, 120-122, 125, 126, 129]  -“Smart Homes” that utilize AI algorithms [61, 62, 110]  -“Nurse Call Systems” that utilize machine learning algorithms [119]  -An “Intelligent ICU” that uses computer vision and deep learning techniques [57]  -AI-driven virtual health care assistant chatbots [20,21,37,38,87]  -“HELPER” fall detection system [128]  - AIHTs used in telehealth [40,118]  -Some articles discussed AIHTs in general [1-3,51,54, 56,60,69, 89, 93, 95-97,100,101, 104, 107, 108] |
| What involvement do nurses have, or are predicted to have, in the co-design of AI-driven digital health technologies? | -Total of 16 articles for this question  -3 studies with quantitative or prototyping methods [62,64,65]  -13 expository papers (including a scoping review and white paper) [1,23,26,41,48,60,63,66, 69,70,105,110,130] | Australia [70]  Canada [66,69]  Germany[23]  Philippines [26]  Singapore[64]  United Kingdom[60]  United States of America [1,41,48,62,63,65,105, 110,130] | Nurses have been involved in the co-design of AI-health technologies in:  -Universities[1,63,64]  -Community settings [1,62,70] | -Most articles discussed the general involvement of nurses in the co-design of AI health technologies[1,26,41, 66,69,105,130]  -Doctorally-prepared nurses, nurse researchers or nurse scientists were often discussed more specifically [48,62,64,65,70,110]  -In the future, it was also predicted that there would be a new discipline of ‘nurse-engineers’ [63]  -Two articles referred to health professionals more generally, but the information was relevant and transferable to nurses [23,60] | Nurses have been involved in the co-design of:  - “Smart Homes” that utilize AI algorithms[62,70, 110]  -AI-driven virtual counseling application that can be used for communication skills training for nursing students[64]  -Robotic technologies [1,63] |

References

1. Robert N. How artificial intelligence is changing nursing. Nurs Manage 2019 Sep; 50(9):30-39. [doi:10.1097/01.NUMA.0000581404.86147.ac]

2. Booth R. Informatics and nursing in a post-nursing informatics world: Future directions for nurses in an automated, artificially-intelligent, social-networked healthcare environment. Canadian Journal of Nursing Leadership 2016; 28 (4): 61–69. [doi: 10.12927/cjnl.2016.24563]

3. Secretary of State for Health and Social Care. The Topol review: Preparing the health care workforce to deliver the digital future [White paper]. 2019. URL: https://topol.hee.nhs.uk/wp-content/uploads/HEE-Topol-Review-2019.pdf [accessed 2020-09-30] 

4. Cramer E M, Seneviratne M G, Sharifi H, Ozturk A, Hernandez-Boussard T. Predicting the incidence of pressure ulcers in the intensive care unit using machine learning. eGEMs (Generating Evidence & Methods to improve patient outcomes) 2019;7 (1):1-11. [doi: 10.5334/egems.307] 

5. Maalouf N, Sidaoui A, Elhajj I H, Asmar D. Robotics in nursing: A scoping review. Journal of Nursing Scholarship 2018; 50 (6): 590–600. [doi: 10.1111/jnu.12424]

6. Tuisku O, Pekkarinen S, Hennala L, Melkas, H. “Robots do not replace a nurse with a beating heart.” The publicity around a robotic innovation in elderly care. Information Technology & People 2019; 32 (1):47–67. [doi: 10.1108/itp-06-2018-0277] 

7. Demange M, Pino M, Kerhervé H, Rigaud A-S, Cantegreil-Kallen I. Management of acute pain in dementia: a feasibility study of a robot-assisted intervention. Journal of Pain Research 2019;12: 1833 –1846. [doi: 10.2147/jpr.s179640] 

8. Jibb LA, Birnie KA, Nathan PC, Beran TN, Hum V, Victor JC, Stinson JN. Using the MEDiPORT humanoid robot to reduce procedural pain and distress in children with cancer: A pilot randomized controlled trial. Pediatric Blood & Cancer 2018; 65 (9):e27242. [doi: 10.1002/pbc.27242]

9. Beran TN, Ramirez-Serrano A, Vanderkooi OG, Kuhn S. Humanoid robotics in health care: An exploration of children’s and parents' emotional reactions. Journal of Health Psychology 2015; 20 (7):984–989. [doi: 10.1177/1359105313504794]

10. Nieto Franco F. Design and implementation of a robotic software for the assessment of pain in children. Studies in Health Technology and Informatics 2018; 250:50–54. [doi: 10.3233/978-1-61499-872-3-50]

11. Tanioka R, Sugimoto H, Yasuhara Y, Ito H, Osaka K, ZhaoY, Kai Y, Locsin R, Tanioka T. Characteristics of transactive relationship phenomena among older adults, care workers as intermediaries, and the Pepper robot with care prevention gymnastics exercises. The Journal of Medical Investigation: JMI 2019; 66(1.2): 46–49. [doi: [10.2152/jmi.66.46](https://doi.org/10.2152/jmi.66.46)]

12. Birks M, Bodak M, Barlas J, Harwood J, Pether M. Robotic seals as therapeutic tools in an aged care facility: A qualitative study. Journal of Aging Research 2016: 1-7. [doi:10.1155/2016/8569602]

13. Liang H-F, Wu K-M, Weng C-H, Hsieh H-W. Nurses’ views on the potential use of robots in the pediatric unit. Journal of Pediatric Nursing 2019; 47 :e58-e64. [doi:10.1016/j.pedn.2019.04.027]

14. Robinson H, Broadbent E, MacDonald B. Group sessions with Paro in a nursing home: Structure, observations and interviews. Australasian Journal on Ageing 2016; 35 (2):106-112. [doi: 10.1111/ajag.12199]

15. Huisman C, Kort H. Two-year use of care robot Zora in Dutch nursing homes: An evaluation study. Healthcare 2019;7 (1): 31.  [doi: 10.3390/healthcare7010031]

16. Bemelmans R, Gelderblom GI, Jonker P, de Witte L. How to use robot interventions in intramural psychogeriatric care: A feasibility study. Applied Nursing Research 2016; 30:154–157. [doi: 10.1016/j.apnr.2015.07.003]

17. Boumans R, van Meulen F, Hindriks K, et al. Robot for health data acquisition among older adults: a pilot randomised controlled cross-over trial. BMJ Quality & Safety 2019; 28: 793-799. [doi: http://dx.doi.org/10.1136/bmjqs-2018-008977]

18. Jones C, Moyle W, Murfield J, Draper B, Shum D, Beattie E, Thalib L. Does cognitive impairment and agitation in dementia influence intervention effectiveness? Findings from a cluster-randomized-controlled trial with the therapeutic robot, PARO. Journal of the American Medical Directors Association 2018; 19 (7): 623-626. [doi:10.1016/j.jamda.2018.02.014]

19. Sicurella T, Fitzsimmons V. Robotic pet therapy in long-term care. Nursing 2016; 46 (6):55–57.  [doi:10.1097/01.NURSE.0000482265.32133.f6]

20. Miller E, Polson D. Apps, avatars, and robots: The future of mental healthcare. Issues in Mental Health Nursing 2019; 40 (3):208-214.[doi:10.1080/01612840.2018.1524535]

21. Luxton DD. Recommendations for the ethical use and design of artificial intelligent care providers. Artificial Intelligence in Medicine 2014; 62 (1):1–10. [doi: 10.1016/j.artmed.2014.06.004]

22. Tanioka T, Yasuhara Y, Dino MJS, Kai Y, Locsin RC, Schoenhofer S. Disruptive engagements with technologies, robotics, and caring: Advancing the transactive relationship theory of nursing. Nurs Admin Q 2019; 43(4):313-321. [doi: 10.1097/NAQ.0000000000000365] 

23. Beck J-P. Are we ready for AI? Why innovation in tech needs to be matched by investment in people. Eurohealth 2019; 25(3). URL: https://apps.who.int/iris/handle/10665/327753 [accessed 2020-08-31] 

24. Kriegel J, Grabner V, Tuttle-Weidinger L, Ehrenmüller I. Socially assistive robots (SAR) in in-patient care for the elderly. Studies in Health Technology and Informatics 2019; 260:178–185. [PMID: 31118335]

25. Vandemeulebroucke T, de Casterlé BD, Welbergen L, Massart M, Gastmans C. The ethics of socially assistive robots in aged care. A focus group study with older adults in Flanders, Belgium. The Journals of Gerontology 2019; 75(9): 1996-2007. [doi:10.1093/geronb/gbz070]

26. Pepito JA, Locsin R. Can nurses remain relevant in a technologically advanced future? International Journal of Nursing Sciences 2019; 6 (1):106-110. [doi:10.1016/j.ijnss.2018.09.013]

27. Tanioka T. Nursing and rehabilitative care of the elderly using humanoid robots. The Journal of Medical Investigation 2019; 66. [doi: 10.2152/jmi.66.19]

28. Gustafsson C, Svanberg C, Müllersdorf M. Using a robotic cat in dementia care: A pilot study. Journal of Gerontological Nursing 2015; 41 (10): 46–56. [doi: 10.3928/00989134-20150806-44]

29. Pfadenhauer M, Dukat C. Robot caregiver or robot-supported caregiving? International Journal of Social Robotics 2015; 7 (3):393–406. [doi: 10.1007/s12369-015-0284-0] 

30. Moyle W, Jones C, Murfield J, Thalib L, Beattie E, Shum D, Draper B. Using a therapeutic companion robot for dementia symptoms in long-term care: reflections from a cluster-RCT. Aging & Mental Health 2019;23 (3):329-336. [doi:10.1080/13607863.2017.1421617]

31. Vandemeulebroucke T, Dierckx de Casterlé B, Gastmans C. The use of care robots in aged care: A systematic review of argument-based ethics literature. Archives of Gerontology and Geriatrics 2018; 74: 15–25. [doi: 10.1016/j.archger.2017.08.014]

32. Zafrani O, Nimrod G. Towards a holistic approach to studying human–robot interaction in later life. The Gerontologist 2019; 59 (1):e26–e36. [doi: 10.1093/geront/gny077]

33. Moyle W, Bramble M, Jones C, Murfield J. Care staff perceptions of a social robot called Paro and a look-alike plush toy: a descriptive qualitative approach. Aging & Mental Health 2018; 22 (3):330–335. [doi: 10.1080/13607863.2016.1262820]

34. Vandemeulebroucke T, de Casterlé BD, Gastmans C. How do older adults experience and perceive socially assistive robots in aged care: a systematic review of qualitative evidence. Aging & Mental Health 2018; 22 (2):149–167. [doi: 10.1080/13607863.2017.1286455]

35. Metzler TA, Lewis LM, Pope LC. Could robots become authentic companions in nursing care? Nursing Philosophy 2016; 17 (1):36–48. [doi: 10.1111/nup.12101]

36. Lynn LA. Artificial intelligence systems for complex decision-making in acute care medicine: a review. Patient Safety in Surgery 2019; 13, 6. [doi: https://doi.org/10.1186/s13037-019-0188-2]

37. Ackerman M, Virani T, Billings B. Digital mental health – Innovations in consumer driven care. Canadian Journal of Nursing Leadership 2017; 30 (3):63–72. [doi: 10.12927/cjnl.2018.25384]

38. Joerin A, Rauws M, Ackerman ML. Psychological artificial intelligence service, Tess: Delivering on-demand support to patients and their caregivers [Technical Report]. Cureus 2019; 11(1): e3972. [doi: 10.7759/cureus.3972]

39. Liao PH, Hsu PT, Chu W, Chu WC. Applying artificial intelligence technology to support decision-making in nursing: A case study in Taiwan. Health Informatics Journal 2015; 21 (2):137–148. [doi: 10.1177/1460458213509806]

40. Kuziemsky C, Maeder AJ, John O, Gogia SB, Basu A, Meher S, Ito M. Role of artificial intelligence within the telehealth domain. Yearbook of Medical Informatics 2019; 28 (1):35-45. [doi: 10.1055/s-0039-1677897] 

41. Clipper B, Batcheller J, Thomaz AL, Rozga A. Artificial intelligence and robotics: A nurse leader’s primer. Nurse Leader 2018; 16 (6):379–384. [doi: 10.1016/j.mnl.2018.07.015] 

42. Archibald MM, Barnard A. Futurism in nursing: Technology, robotics and the fundamentals of care. Journal of Clinical Nursing 2018; 27 (11-12): 2473–2480. [doi: 10.1111/jocn.14081]

43. Papadopoulos I, Koulouglioti C, Ali S. Views of nurses and other health and social care workers on the use of assistive humanoid and animal-like robots in health and social care: a scoping review. Contemporary Nurse 2018; 54 (4-5):425–442. [doi: 10.1080/10376178.2018.1519374]

44. Glauser W. Artificial intelligence, automation and the future of nursing. The Canadian Nurse 2017; 113 (3):24–26. [PMID: 29235787]

45. Ding M, Matsubara T, Funaki Y, Ikeura R, Mukai T, Ogasawara T. Generation of comfortable lifting motion for a human transfer assistant robot. International Journal of Intelligent Robotics and Applications 2017; 1 (1):74–85. [doi: 10.1007/s41315-016-0009-z] 

46. Gannod GC, Abbott KM, Van Haitsma K, Martindale N, Heppner A. A machine learning recommender system to tailor preference assessments to enhance person-centered care among nursing home residents. The Gerontologist 2019; 59 (1):167–176. [doi: 10.1093/geront/gny056]

47. McGrow K. Artificial intelligence: Essentials for nursing. Nursing 2019; 49(9), 46–49. [doi: 10.1097/01.NURSE.0000577716.57052.8d]

48. Linnen DT, Javed PS, DʼAlfonso JN. Ripe for disruption? Adopting nurse-led data science and artificial intelligence to predict and reduce hospital-acquired outcomes in the learning health system. Nursing Administration Quarterly 2019; 43 (3):246–255. [doi: 10.1097/NAQ.0000000000000356]

49. Olling K, Nyeng DW, Wee L. Predicting acute odynophagia during lung cancer radiotherapy using observations derived from patient-centred nursing care. Technical Innovations & Patient Support in Radiation Oncology 2018; 5: 16–20. [doi: 10.1016/j.tipsro.2018.01.002] 

50. Mandal I. Machine learning algorithms for the creation of clinical healthcare enterprise systems. Enterprise Information Systems 2016: 1–27. [doi: 10.1080/17517575.2016.1251617] 

51. Health Education England. (2018). The Topol review interim report: Preparing the health care workforce to deliver the digital future [White paper]. https://www.hee.nhs.uk/sites/default/files/documents/Topol%20Review%20interim%20report_.pdf [accessed 2020-08-31]

52. HIMSS. 2019. Artificial intelligence, critical thinking and the nursing process. URL: https://www.himss.org/library/artificial-intelligence-critical-thinking-and-nursing-process [accessed 2020-08-31] 

53. HIMSS. 2018. AI and nursing impact on the quadruple aim. URL: https://www.himss.org/news/ai-and-nursing-impact-quadruple-aim [accessed 2020-08-31] 

54. NHSX. 2019. Artificial Intelligence: How to get it right [White paper]. URL: https://www.nhsx.nhs.uk/media/documents/NHSX_AI_report.pdf [accessed 2020-08-31] 

55. Li H-L, Lin, S-W, Hwang, Y-T. Using nursing information and data mining to explore the factors that predict pressure injuries for patients at the end of life. Computers, Informatics, Nursing: CIN 2019; 37 (3):133–141. [doi: 10.1097/CIN.0000000000000489]

56. Sensmeier J. Harnessing the power of artificial intelligence. Nursing Management 2017; 48 (11):14–19. [doi: 10.1097/01.NUMA.0000526062.69220.41]

57. Davoudi A, Malhotra KR, Shickel B, Siegel S, Williams S, Ruppert M, Bihorac E, Ozrazgat-Baslanti T, Tighe PJ, Bihorac A, Rashidi P. Intelligent ICU for autonomous patient monitoring using pervasive sensing and deep learning. Scientific Reports 2019; 9 (1):8020. [doi: 10.1038/s41598-019-44004-w]

58. Byrne MD. Machine learning in health care. Journal of PeriAnesthesia Nursing 2017; 32 (5):494-496 [doi: 10.1016/j.jopan.2017.07.004]

59. Salzmann-Erikson M, Eriksson H. Letter to the editor: Prosperity of nursing care robots: an imperative for the development of new infrastructure and competence for health professions in geriatric care. Journal of Nursing Management 2017; 25 (6):486–488. [doi: 10.1111/jonm.12487]

60. Foley T, Wollard J. Health Education England. 2019. The digital future of mental healthcare and its workforce: A report on a mental health stakeholder engagement to inform the Topol Review [White paper]. URL: https://topol.hee.nhs.uk/wp-content/uploads/HEE-Topol-Review-Mental-health-paper.pdf [accessed 2020-08-31] 

61. Fritz RL, Corbett CL, Vandermause R, Cook D. The influence of culture on older adults’ adoption of smart home monitoring. Gerontechnology 2016;14 (3) [doi: 10.4017/gt.2016.14.3.010.00] 

62. Fritz RL, Dermody G. A nurse-driven method for developing artificial intelligence in “smart” homes for aging-in-place. Nursing Outlook 2019; 67 (2):140–153. [doi: 10.1016/j.outlook.2018.11.004]

63. Glasgow MES, Colbert A, Viator J, Cavanagh S. The nurse‐engineer: A new role to improve nurse technology interface and patient care device innovations. Journal of Nursing Scholarship 2018; 50 (6):601–611. [doi: 10.1111/jnu.12431]

64. Shorey S, Ang E, Yap J, Ng ED, Lau ST, Chui CK. A virtual counseling application using artificial intelligence for communication skills training in nursing education: Development study. Journal of Medical Internet Research 2019; 21 (10), e14658. [doi: 10.2196/14658] 

65. Backonja U, Hall AK, Painter I, Kneale L, Lazar A, Cakmak M, Thompson JH, Demiris G. Comfort and attitudes towards robots among young, middle-aged, and older adults: A cross-sectional study. Journal of Nursing Scholarship 2018; 50 (6):623–633. [doi: 10.1111/jnu.12430]

66. Risling T. 2018. Why AI needs nursing. Policy Options. URL: https://policyoptions.irpp.org/magazines/february-2018/why-ai-needs-nursing/ [accessed 2020-08-31] 

67. Papadopoulos I, Koulouglioti C. The influence of culture on attitudes towards humanoid and animal‐like robots: An integrative review. Journal of Nursing Scholarship 2018; 50 (6):653–665. [doi: 10.1111/jnu.12422]

68. Carroll WM. The synthesis of nursing knowledge and predictive analytics. Nursing Management 2019; 50 (3):15-17. [doi: 10.1097/01.numa.0000553503.78274.f7] 

69. Risling TL, Low C. Advocating for safe, quality and just care: What nursing leaders need to know about artificial intelligence in healthcare delivery. Nursing Leadership 2019; 32 (2):31–45. [doi: 10.12927/cjnl.2019.25963]

70. Dermody G, Fritz R. A conceptual framework for clinicians working with artificial intelligence and health-assistive Smart Homes. Nursing Inquiry 2019; 26 (1): e12267. [doi: 10.1111/nin.12267]

71. Klein B, Schlömer I. A robotic shower system: Acceptance and ethical issues. Zeitschrift Fur Gerontologie Und Geriatrie 2018; 51 (1):25–31. [doi: 10.1007/s00391-017-1345-9]

72. Fiorini L, De Mul M, Fabbricotti I, Limosani R, Vitanza A, D’Onofrio G, et al. Assistive robots to improve the independent living of older persons: results from a needs study. Disability and Rehabilitation: Assistive Technology 2019: 1–11. [doi: 10.1080/17483107.2019.1642392] 

73. Law M, Sutherland C, Ahn HS, MacDonald BA, Peri K, Johanson DL, et al. Developing assistive robots for people with mild cognitive impairment and mild dementia: a qualitative study with older adults and experts in aged care. BMJ Open 2019; 9 (9): e.031937. [doi: 10.1136/bmjopen-2019-031937] 

74. Poulsen A, Burmeister OK. Overcoming carer shortages with care robots: Dynamic value trade-offs in run-time. Australasian Journal of Information Systems 2019; 23. [doi: 10.3127/ajis.v23i0.1688] 

75. Lee H, Piao M, Lee J, Byun A, Kim J. The purpose of bedside robots: exploring the needs of inpatients and healthcare professionals. CIN: Computers, Informatics, Nursing 2020; 38 (1):8-17. [doi: 10.1097/CIN.0000000000000558]

76. Coco K, Kangasniemi M, Rantanen T. Care personnel’s attitudes and fears toward care robots in elderly care: A comparison of data from the care personnel in Finland and Japan. Journal of Nursing Scholarship 2018; 50(6):634–644. [doi: 10.1111/jnu.12435]

77. Rantanen T, Lehto P, Vuorinen P, Coco K. The adoption of care robots in home care—A survey on the attitudes of Finnish home care personnel. Journal of Clinical Nursing 2018; 27 (9-10):1846–1859. [doi: 10.1111/jocn.14355]

78. Bemelmans R, Gelderblom GJ, Jonker P, de Witte L. Effectiveness of robot Paro in intramural psychogeriatric care: A multicenter quasi-experimental study. Journal of the American Medical Directors Association 2015; 16 (11):946–950. [doi: 10.1016/j.jamda.2015.05.007]

79. Chen TL, King C-HA, Thomaz AL, Kemp CC. An investigation of responses to robot-initiated touch in a nursing context. International Journal of Social Robotics 2014; 6 (1):141–161. [doi:10.1007/s12369-013-0215-x] 

80. de Fátima Fernandes MN, Esteves RB, Teixeira CAB, da Silva Gherardi-Dona EC. The present and the future of nursing in the brave new world. Revista Da Escola de Enfermagem Da U S P 2018; 52: e03356. [doi: 10.1590/S1980-220X2017031603356]

81. Nwosu AC, Collins B, Mason S. Big Data analysis to improve care for people living with serious illness: The potential to use new emerging technology in palliative care. Palliative Medicine 2018; 32 (1):164–166. [doi: 10.1177/0269216317726250]

82. Moyle W, Jones C, Pu L, Chen S-C. Applying user-centred research design and evidence to develop and guide the use of technologies, including robots, in aged care. Contemporary Nurse 2018; 54 (1):1–3. [doi: 10.1080/10376178.2017.1438057]

83. Nairn S. On being a Luddite in the new world of technological nursing care. Nursing Philosophy 2016; 7 (1):3-5. [doi: 10.1111/nup.12116]

84. Whelton BJB. Being human in a global age of technology. Nursing Philosophy 2016; 17 (1):28-35. [doi: 10.1111/nup.12109]

85. Monteiro APTdAV. Cyborgs, biotechnologies, and informatics in health care--new paradigms in nursing sciences. Nursing Philosophy 2016; 17 (1):19–27. [doi: 10.1111/nup.12088]

86. Newland J. Humans versus artificial intelligence. The Nurse Practitioner 2015; 40 (9): 13. [doi: 10.1097/01.NPR.0000470365.61826.77]

87. Hernandez JPT. Network diffusion and technology acceptance of a nurse chatbot for chronic disease self-management support: A theoretical perspective. The Journal of Medical Investigation 2019: 66, 24-30. [doi:10.2152/jmi.66.24]

88. Gallagher A, Nåden D, Karterud D. Robots in elder care: Some ethical questions. Nursing Ethics 2016; 23 (4): 369–371. [doi: 10.1177/0969733016647297] 

89. Alder Hey Children’s NHS Foundation Trust (n.d.). Digital Futures. [White paper]. URL: https://alderhey.nhs.uk/application/files/2215/6450/2932/Digital_Futures.pdf [accessed 2020-08-31] 

90. Clavelle JT, Sweeney CD, Swartwout E, Lefton C, Guney S. Leveraging technology to sustain extraordinary care: A qualitative analysis of meaningful nurse recognition. The Journal of Nursing Administration 2019; 49 (6):303–309. [doi: 10.1097/NNA.0000000000000757]

91. Poncette A-S, Spies C, Mosch L, Schieler M, Weber-Carstens S, Krampe H, et al. Clinical requirements of future patient monitoring in the intensive care unit: Qualitative study. JMIR Medical Informatics 2019; 7 (2): e13064. [doi: 10.2196/13064]

92. Ujike S, Yasuhara Y, Osaka K, Sato M, Catangui E, Edo S, et al. Encounter of Pepper-CPGE for the elderly and patients with schizophrenia: an innovative strategy to improve patient’s recreation, rehabilitation, and communication. The Journal of Medical Investigation 2019; 66: 50-5.[doi:10.2152/jmi.66.50]

93. Morley J, Joshi I. Developing effective policy to support artificial intelligence in health and care. Eurohealth 2019; 25 (‎2)‎. URL: https://apps.who.int/iris/handle/10665/326127 [accessed 2020-08-21] 

94. Carroll W. Predicting severe maternal morbidity and mortality - An informatics opportunity. Online Journal of Nursing Informatics 2018; 22 (3). URL: https://www.himss.org/library/predicting-severe-maternal-morbidity-and-mortality-informatics-opportunity [accessed 2020-08-31] 

95. Carroll W. Nursing informaticists safeguarding the use of emerging technologies. Online Journal of Nursing Informatics 2019; 23 (3). URL: https://www.himss.org/resources/nursing-informaticists-safeguarding-use-emerging-technologies [accessed 2020-08-31] 

96. Kaminski J. Exploring the nationwide interoperability roadmap from a nursing perspective. Online Journal of Nursing Informatics 2018; 22 (2). URL: https://www.himss.org/library/exploring-nationwide-interoperability-roadmap-nursing-perspective [accessed 2020-08-31] 

97. Meetoo D, Rylance R. AI: revolution or apocalypse? The British Journal of Nursing 2018; 27 (19):1092–1092. [doi: 10.12968/bjon.2018.27.19.1092]

98. Carter‐Templeton H, Frazier RM, Wu L, H Wyatt T. Robotics in nursing: A bibliometric analysis. Journal of Nursing Scholarship 2018; 50 (6): 582–589. [doi: 10.1111/jnu.12399]

99. Sherry J. The robot nurses are coming to a workplace near you. The British Journal of Nursing 2018; 27 (13):765–767. [doi: 10.12968/bjon.2018.27.13.765]

100. Ganapathy K, Abdul SS, Nursetyo AA. Artificial intelligence in neurosciences: A clinician’s perspective. Neurology India 2018; 66 (4):934–939. [10.4103/0028-3886.236971]

101. Skiba DJ. Augmented intelligence and nursing. Nursing Education Perspectives 2017; 38 (2):108–109. [doi:10.1097/01.NEP.0000000000000124]

102. Paulson SS, Scruth E. Legal and ethical concerns of big data: Predictive analytics. Clinical Nurse Specialist 2017; 31 (5):237–239. [doi: 10.1097/NUR.0000000000000315]

103. Ishiguro K, Majima Y. Utilization of communication robot in patient education. Nursing Informatics 2016; 225: 913-914. [doi:10.3233/978-1-61499-658-3-913] 

104. Carrière R, MacDonald A, Chan Y. Past, present and future: The outlook from mid-career nurse informaticians. Nursing Leadership 2016; 28 (4):8–17. [doi: 10.12927/cjnl.2016.24556]

105. Sharts-Hopko NC. The coming revolution in personal care robotics: What does it mean for nurses? Nursing Administration Quarterly 2014; 38 (1): 5-12. [doi: 10.1097/NAQ.0000000000000000]

106. Frazier RM, Carter-Templeton H, Wyatt TH, Wu L. Current trends in robotics in nursing patents—A glimpse into emerging innovations. CIN: Computers, Informatics, Nursing 2019; 37 (6):290-297. [doi: 10.1097/CIN.0000000000000538]

107. Peirce AG, Elie S, George A, Gold M, O’Hara K, Rose-Facey W. Knowledge development, technology and questions of nursing ethics. Nursing Ethics 2020; 27 (1): 77-87. [doi: 10.1177/0969733019840752] 

108. Perry L. Machine learning: Great opportunities, but will it replace nurses? International Journal of Nursing Practice 2019; 25 (1): e12725. [doi: 10.1111/ijn.12725] 

109. Woods JS, Saxena M, Nagamine T, Howell RS, Criscitelli T, Gorenstein S, et al. The future of data-driven wound care. AORN Journal 2018; 107 (4):455–463. [doi: 10.1002/aorn.12102]

110. Nguyen-Truong CKY, Fritz RL. Health-assistive smart homes for aging in place: Leading the way for integration of the Asian immigrant minority voice. Asian/Pacific Island Nursing Journal 2018; 3 (4):154–159. [doi: 10.31372/20180304.1087] 

111. Kim J. Use of robots as a creative approach in healthcare ICT. Healthcare Informatics Research 2018; 24 (3):155. [doi: 10.4258/hir.2018.24.3.155] 

112. Effken JA. Issues, impacts and insights column: What’s new in healthcare robotics? Online Journal of Nursing Informatics 2014; 18 (3):1. 

113. Jamieson T, Goldfarb A. Clinical considerations when applying machine learning to decision-support tasks versus automation. BMJ Quality & Safety 2019; 28 (10):778–781. [doi: 10.1136/bmjqs-2019-009514] 

114. Lo Y, Lynch SF, Urbanowicz RJ, Olson RS, Ritter AZ, Whitehouse CR, et al. Using machine learning on home health care assessments to predict fall risk. Studies in Health Technology and Informatics 2019; 264: 684–688. [doi: 10.3233/SHTI190310]

115. Kwon JY, Karim ME, Topaz M, Currie LM. Nurses “seeing forest for the trees” in the age of machine learning: Using nursing knowledge to improve relevance and performance. Computers, Informatics, Nursing: CIN 2019; 37 (4):203-212. [doi:10.1097/CIN.0000000000000508]

116. Guidi G, Pollonini L, Dacso CC, Iadanza E. A multi-layer monitoring system for clinical management of congestive heart failure. BMC Medical Informatics and Decision Making 2015; 15 (Suppl 3): S5. [doi: 10.1186/1472-6947-15-S3-S5]

117. Sikka K, Ahmed AA, Diaz D, Goodwin MS, Craig KD, Bartlett MS, et al. Automated assessment of children’s postoperative pain using computer vision. Pediatrics 2015; 136 (1): e124–e131. [doi: 10.1542/peds.2015-0029]

118. Lin H, Hsu Y-L, Hsu M-S, Cheng C-M. Development of a telehealthcare decision support system for patients discharged from the hospital. Telemedicine Journal and E-Health 2014; 20 (8):748–756. [doi: 10.1089/tmj.2013.0261]

119. Ongenae F, Claeys M, Kerckhove W, Dupont T, Verhoeve P, De Turck F. A self-learning nurse call system. Computers in Biology and Medicine 2014;44: 110-123. [doi: 10.1016/j.compbiomed.2013.10.014]

120. Park JI, Bliss DZ, Chi C-L, Delaney CW, Westra BL. Knowledge discovery with machine learning for hospital-acquired catheter-associated urinary tract infections. CIN: Computers, Informatics, Nursing 2020; 38 (1): 28-35. [doi: 10.1097/CIN.0000000000000562]

121. Ginestra JC, Giannini HM, Schweickert WD, Meadows L, Lynch MJ, Pavan K, et al. Clinician perception of a machine learning–based early warning system designed to predict severe sepsis and septic shock. Critical Care Medicine 2019; 47 (11):1477-1484. [doi: 10.1097/ccm.0000000000003803] 

122. Sullivan SS, Hewner S, Chandola V, Westra BL. Mortality risk in homebound older adults predicted from routinely collected nursing data. Nursing Research 2019; 68 (2):156–166. [doi: 10.1097/nnr.0000000000000328] 

123. Lee J-Y, Song YA, Jung JY, Kim HJ, Kim BR, Do H-K, Lim J-Y. Nurses’ needs for care robots in integrated nursing care services. Journal of Advanced Nursing 2018; 74 (9): 2094-2105. [doi: 10.1111/jan.13711 ] 

124. Turja T, Van Aerschot L, Särkikoski T, Oksanen A. Finnish healthcare professionals’ attitudes towards robots: Reflections on a population sample. Nursing Open 2018; 5 (3):300–309. [doi: 10.1002/nop2.138] 

125. Lodhi MK, Stifter J, Yao Y, Ansari R, Keenan GM, Wilkie DJ, Khokhar AA. Predictive modeling for end-of-life pain outcome using electronic health records. Advanced Data Mining 2015: 56-68. [doi:10.1007/978-3-319-20910-4_5] 

126. Sparks RS, Okugami C. Tele-Health monitoring of patient wellness. Journal of Intelligent Systems 2016; 25 (4). [doi: 10.1515/jisys-2014-0175] 

127. Nauta J, Mahieu C, Michiels C, Ongenae F, De Backere F, De Turck F, et al. Pro-active positioning of a social robot intervening upon behavioral disturbances of persons with dementia in a smart nursing home. Cognitive Systems Research 2019; 57:160–174. [doi: 10.1016/j.cogsys.2019.03.002] 

128. Coahran M, Hillier LM, Van Bussel L, Black E, Churchyard R, Gutmanis I, et al. Automated fall detection technology in inpatient geriatric psychiatry: Nurses’ perceptions and lessons learned. Canadian Journal on Aging 2018; 37 (3):245–260. [doi: 10.1017/S0714980818000181]

129. Menon U, Cohn E, Downs CA, Gephart SM, Redwine L. Precision health research and implementation reviewed through the conNECT framework. Nursing Outlook 2019; 67 (4):302–310. [doi: 10.1016/j.outlook.2019.05.010]

130. Frith KH. Artificial intelligence: What does it mean for nursing? Nursing Education Perspectives 2019; 40 (4):261. [doi: 10.1097/01.NEP.0000000000000543]

131. Locsin RC. The co-existence of technology and caring in the theory of technological competency as caring in nursing. The Journal of Medical Investigation 2017; 64(1.2): 160–164. [doi: 10.2152/jmi.64.160]
