# Supplementary material for: Predicted Influences of Artificial Intelligence on the Domains of Nursing: Scoping Review
Source: JMIR Nurs. 2020 Dec 17;3(1):e23939. doi: 10.2196/23939 (PMC8373374; doi:10.2196/23939)
Supplement: Supplementary file 3 [file nursing_v3i1e23939_app1.docx]

Search strategy: 2019-10-18 HS - DRAFT 8 - AI and Nurses and 10 yrs or 5 yrs - Medl

Topic: Artificial Intelligence and Nurses/Nursing
Database: Ovid MEDLINE(R) 1946 to October 17 2019
Date searched: Friday, October 18, 2019

2019-10-18 HS - DRAFT 8 - AI and Nurses and 10 yrs or 5 yrs - Medl

Post-teleconference discussion changes

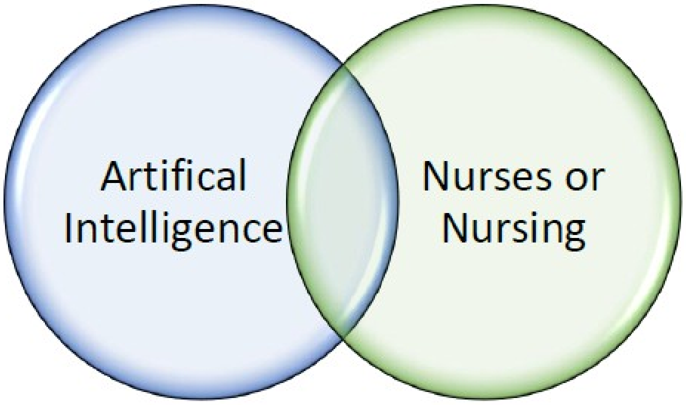

Medline

Ovid MEDLINE(R) 1946 to October 17, 2019

#	Searches	Results
1	Artificial Intelligence/	22239
2	exp Automation/	38969
3	Big Data/	543
4	Biomedical Technology/	6215
5	Computer Heuristics/	22
6	Computer-Aided Design/	14862
7	Data Aggregation/	13
8	Data Analysis/	784
9	Data Mining/	7741
10	Data Science/	105
11	Data Warehousing/	92
12	Decision Making, Computer-Assisted/	2727
13	Deep Learning/	978
14	Diagnosis, Computer-Assisted/	22053
15	Drug Therapy, Computer-Assisted/	1653

Search strategy: 2019-10-18 HS - DRAFT 8 - AI and Nurses and 10 yrs or 5 yrs - Medl

Topic: Artificial Intelligence and Nurses/Nursing
Database: Ovid MEDLINE(R) 1946 to October 17 2019
Date searched: Friday, October 18, 2019

16	Expert Systems/	3351
17	Fuzzy Logic/	4331
18	Information Theory/	3836
19	Knowledge Bases/	1374
20	Language/ and Translating/	1081
21	Latent Class Analysis/	295
22	Machine Learning/	6877
23	medical informatics applications/	2447
24	medical informatics/	11417
25	multifactor dimensionality reduction/	189
26	Natural Language Processing/	3970
27	"Neural Networks (Computer)"/	23434
28	Numerical Analysis, Computer-Assisted/	5507
29	Pattern Recognition, Automated/	24734
30	Radiographic Image Interpretation, Computer-Assisted/	13848
31	Robotics/	19648
32	Signal Processing, Computer-Assisted/	45572
33	Supervised Machine Learning/	436
34	Support Vector Machine/	5784
35	Therapy, Computer-Assisted/	6598
36	Unsupervised Machine Learning/	231
37	"big data".mp.	3734
38	(ai and intelligence).tw.	450
39	(algor#th* adj2 (computer* or machine? or intelligen* or art#ficial* or analy*)).mp.	9550
40	(art#ficial* adj2 intelligen*).mp.	23805
41	(automat* adj2 algor#th*).mp.	3798
42	(automat* adj2 pattern? adj2 analy*).mp.	39
43	(automat* adj2 pattern? adj2 recog*).mp.	24792
44	(automat*4 and (computer* or technolog*)).mp.	69308
45	(comput* adj1 analy*).mp.	19252
46	(comput* adj2 pattern? adj2 analy*).mp.	60
47	(comput* adj2 pattern? adj2 recog*).mp.	129
48	(comput* adj2 pattern? adj2 recog*).mp.	129
49	(comput* adj2 reasoning*).mp.	64
50	(computat* adj2 intelligen*).mp.	245

Search strategy: 2019-10-18 HS - DRAFT 8 - AI and Nurses and 10 yrs or 5 yrs - Medl

Topic: Artificial Intelligence and Nurses/Nursing
Database: Ovid MEDLINE(R) 1946 to October 17 2019
Date searched: Friday, October 18, 2019

51	(computational adj1 approach??).mp.	6658
52	(computational adj1 repurpos*).mp.	6
53	(computer* adj1 vision* adj1 system?).mp.	137
54	(computer* adj2 heuristic*).mp.	26
55	(connectionist* adj2 model*).mp.	408
56	(data adj1 mining).mp.	12292
57	(deep* adj2 learn*).mp.	2647
58	(digital adj2 technolog*).mp.	1696
59	(ensemble adj1 model*).mp.	503
60	(expert* adj1 system?).mp.	4716
61	(fuzzy adj2 logic*).mp.	4618
62	(generali* adj1 boost?? model*).mp.	35
63	(hierarchic* adj1 learn*).mp.	51
64	(high adj1 throughput?).mp.	93965
65	(inductive* adj1 machine* adj1 learn*).mp.	21
66	(information?? adj1 technolog*).mp.	10714
67	(knowledge acqui* and computer*).mp.	380
68	(knowledge base? and computer).mp.	2442
69	(knowledge represent* and computer).mp.	359
70	(learning adj1 model* adj1 algorithm*).mp.	3
71	(learning adj2 algorithm*).mp.	4545
72	(machine? adj2 assist* adj2 discover*).mp.	0
73	(machine? adj2 intelligen*).mp.	198
74	(machine? adj2 learn*).mp.	16965
75	(natural* adj2 language? adj2 process*).mp.	4614
76	((neural* adj2 network*) and computer).mp.	26594
77	(neural* adj2 network* adj2 model*).mp.	3607
78	(predictive adj2 algor#th*).mp.	805
79	(predictive adj2 analy*).mp.	3002
80	(structural equation* adj2 model*).mp.	12873
81	(support vector? adj1 machine?).mp.	11669
82	(support vector? adj1 network*).mp.	88
83	((text? or textual*) adj2 analy*).mp.	2065
84	art#ficial neural network*.mp.	7705
85	big health* data.mp.	21

Search strategy: 2019-10-18 HS - DRAFT 8 - AI and Nurses and 10 yrs or 5 yrs - Medl

Topic: Artificial Intelligence and Nurses/Nursing
Database: Ovid MEDLINE(R) 1946 to October 17 2019
Date searched: Friday, October 18, 2019

86	digital future?.mp.	26
87	digital health*.mp.	651
88	digital intervention?.mp.	128
89	ehealth*.mp.	2143
90	image recognition.mp.	326
91	intelligent machine?.mp.	53
92	knowledgebase?.mp.	641
93	latent class analys#s.mp.	2936
94	latent class model*.mp.	614
95	latent semantic analys#s.mp.	178
96	latent variable model*.mp.	595
97	learning from labeled data.mp.	0
98	machine assisted discovery.mp.	0
99	machine-assist* discover*.mp.	0
100	mhealth*.mp.	1876
101	mobile health*.mp.	5489
102	natural speech processing.mp.	8
103	perceptron?.mp.	1577
104	robotic?.mp.	30900
105	self-learning.mp.	651
106	semisupervised learning.mp.	54
107	semi-supervised learning.mp.	225
108	supervised machine* learn*.mp.	941
109	autonomous.mp. [Added Oct 16 2019]	27357
110	clinical analytic?.mp. [Added Oct 16 2019]	47
111	cognitive computing.mp. [Added Oct 16 2019]	37
112	cognitive technolog*.mp. [Added Oct 16 2019]	23
113	intelligent technolog*.mp. [Added Oct 16 2019]	33
114	(robot or robots).mp. [Added Oct 16 2019]	13807
115	smart technolog*.mp. [Added Oct 16 2019]	125
116	or/1-115 [Artificial Intelligence or Machine Learning & related terms]	472968
117	exp Nurses/	86092
118	exp Nursing/	249142
119	Advanced Practice Nursing/	1576
120	exp Education, Nursing/	82193

Search strategy: 2019-10-18 HS - DRAFT 8 - AI and Nurses and 10 yrs or 5 yrs - Medl

Topic: Artificial Intelligence and Nurses/Nursing
Database: Ovid MEDLINE(R) 1946 to October 17 2019
Date searched: Friday, October 18, 2019

121	exp Nursing Assessment/	32358
122	exp Nursing Care/	132949
123	exp Nursing Process/	83510
124	exp Nursing Staff/	64813
125	Legislation, Nursing/	3137
126	Licensed Practical Nurses/	65
127	Licensure, Nursing/	4573
128	Models, Nursing/	11920
129	Nurse's Role/	40172
130	Nursing Administration Research/	2347
131	Nursing Audit/	3002
132	Nursing Informatics/	1482
133	Nursing Services/	4083
134	Nursing Staff, Hospital/	44181
135	Nursing Stations/	58
136	Nursing, Practical/	3434
137	Nursing, Team/	2518
138	Philosophy, Nursing/	6329
139	Shared Governance, Nursing/	5
140	Societies, Nursing/	21893
141	Students, Nursing/	23639
142	Transcultural Nursing/	3354
143	nurs*.mp,kw.	671984
144	nu.fs.	131984
145	nurs*.jw.	466209
146	nurs*.in.	169910
147	or/117-146 [Nurses / Nursing]	860405
**148**	**116 and 147 [AI + Nursing]**	**11735**
149	limit 148 to english language	10853
**150**	**limit 149 to yr="2010 -Current"**	**6156**
**151**	**limit 149 to yr="2014 -Current"**	**4511**

First 100 resulting citations provided in EndNote format for review.
